# Genetic Diversity of *norA*, Coding for a Main Efflux Pump of *Staphylococcus aureus*

**DOI:** 10.3389/fgene.2018.00710

**Published:** 2019-01-09

**Authors:** Sofia Santos Costa, Benjamin Sobkowiak, Ricardo Parreira, Jonathan D. Edgeworth, Miguel Viveiros, Taane G. Clark, Isabel Couto

**Affiliations:** ^1^Global Health and Tropical Medicine, Instituto de Higiene e Medicina Tropical, Universidade NOVA de Lisboa, Lisbon, Portugal; ^2^Faculty of Infectious and Tropical Diseases, London School of Hygiene and Tropical Medicine, London, United Kingdom; ^3^Department of Infectious Diseases, Centre for Clinical Infection and Diagnostics Research, Guy’s and St Thomas’ NHS Foundation Trust, King’s College London, London, United Kingdom; ^4^Faculty of Epidemiology and Population Health, London School of Hygiene and Tropical Medicine, London, United Kingdom

**Keywords:** *Staphylococcus aureus*, *norA*, alleles, variability, efflux

## Abstract

NorA is the best studied efflux system of *Staphylococcus aureus* and therefore frequently used as a model for investigating efflux-mediated resistance in this pathogen. NorA activity is associated with resistance to fluoroquinolones, several antiseptics and disinfectants and several reports have pointed out the role of efflux systems, including NorA, as a first-line response to antimicrobials in *S. aureus*. Genetic diversity studies of the gene *norA* have described three alleles; *norAI*, *norAII* and *norAIII*. However, the epidemiology of these alleles and their impact on NorA activity remains unclear. Additionally, increasing studies do not account for *norA* variability when establishing relations between resistance phenotypes and *norA* presence or reported absence, which actually corresponds, as we now demonstrate, to different *norA* alleles. In the present study we assessed the variability of the *norA* gene present in the genome of over 1,000 *S. aureus* isolates, corresponding to 112 *S. aureus* strains with whole genome sequences publicly available; 917 MRSA strains sourced from a London-based study and nine MRSA isolates collected in a major Hospital in Lisbon, Portugal. Our analyses show that *norA* is part of the core genome of *S. aureus.* It also suggests that occurrence of *norA* variants reflects the population structure of this major pathogen. Overall, this work highlights the ubiquitous nature of *norA* in *S. aureus* which must be taken into account when studying the role played by this important determinant on *S. aureus* resistance to antimicrobials.

## Introduction

*Staphylococcus aureus* is one of the major human pathogens in the hospital and community settings, causing a wide array of clinical manifestations, from mild skin infections to life-threatening systemic infections (Chambers and DeLeo, 2009; Tong et al., 2015). Development and acquisition of resistance to antibiotics and other antimicrobials is of paramount importance in *S. aureus*, as exemplified by the common occurrence and dissemination of strains displaying a phenotype of multidrug resistance (MDR), including methicillin-resistant *Staphylococcus aureus* (MRSA) strains (Chambers and DeLeo, 2009). MRSA are spread worldwide and are a public health threat, ranked amongst the major nosocomial pathogens (Lee et al., 2018; Tacconelli et al., 2018). MRSA epidemiology has revealed the local or global dissemination of a few number of clones, namely the lineages in clonal complexes CC1, CC5, CC8, CC22, CC30, CC45, CC59, and CC80 (Lee et al., 2018).

In recent years, several studies have supported drug efflux as a player in the emergence of resistance toward antibiotics and other antimicrobials in *S. aureus* (DeMarco et al., 2007; Furi et al., 2013; Kwak et al., 2013; Costa et al., 2015). Of particular interest are multidrug efflux pumps (MDR EPs), which extrude a wide range of chemically dissimilar antimicrobials, being frequently associated with MDR phenotypes in bacteria (Piddock, 2006; Poole, 2007). In *S. aureus*, more than twenty putative MDR EPs are encoded in the chromosome (Schindler et al., 2015), of which several have already been characterized (Costa et al., 2013b). Among these, NorA is the most well studied, being frequently used as a model for studying efflux-mediated resistance in this pathogen.

NorA is a 388 aminoacid protein with 12 transmembrane segments (TMS) that belongs to the Major Facilitator Superfamily (MFS) of secondary transporters. This MDR EP uses the proton motive force to extrude from the cell fluoroquinolones, ethidium bromide, quaternary ammonium compounds, and other antimicrobials (Yoshida et al., 1990; Kaatz et al., 1993; Neyfakh et al., 1993). Several reports have associated NorA activity to low-level resistance to fluoroquinolones and reduced susceptibility to biocides (DeMarco et al., 2007; Huet et al., 2008; Kosmidis et al., 2010; Costa et al., 2011, 2015; Furi et al., 2013). Other studies support a broader substrate range for NorA, including siderophores (Deng et al., 2012) and fusaric acid (Marchi et al., 2015).

The NorA encoding gene, *norA*, was first identified in the chromosome of a fluoroquinolone-resistant *S. aureus* isolate, collected in 1986 at a Japanese hospital (Ubukata et al., 1989). Early studies have shown that genetic diversity of this gene can be captured by three alleles, differing up to 10% at the level of the nucleotide sequence and 5% in the polypeptide sequence; *norAI* (Yoshida et al., 1990), *norAII* (also described as *norA23*) (Noguchi et al., 2004) and *norAIII* (also described as *norA1199*) (Kaatz et al., 1993). The occurrence of *norA* variants is also strengthened by a recent study by Brooks et al. (2018) that refers to the variability of this gene in a set of over 150 *S. aureus* strains. Although some studies have been conducted to ascertain the impact of this genetic diversity on the efflux activity of NorA (Schmitz et al., 1998; Sierra et al., 2000; Noguchi et al., 2004), this effect remains unclear.

Despite this early characterization, there are still contradictory reports in literature on the role of NorA in *S. aureus* efflux-mediated antimicrobial resistance. Several studies have reported on the putative absence of *norA*, most probably due to failure to amplify this gene with primers directed to only one of the possible *norA* alleles. The present study aims at clarifying some of these aspects, by demonstrating that the *norA* gene is part of the core genome of *S. aureus*, reflecting on the genetic variability of the gene, its distribution amongst *S. aureus* clonal lineages, including both methicillin-resistant and -susceptible strains and possible impact on NorA function.

## Materials and Methods

### Study Datasets

Four sets of nucleotide sequences of the *norA* structural gene and the corresponding polypeptide sequences were used in this study, comprising (i) the sequences of the three *norA* alleles described to date in literature; (ii) the *norA* sequences from 112 *S. aureus* whole genome sequences retrieved from the GenBank database; (iii) the *norA* sequences from 917 MRSA strains sourced from a London-based study (Auguet et al., 2018) (ENA accession PRJEB11177); (iv) the *norA* sequences of nine MRSA isolates collected in a major Hospital in Lisbon, Portugal, representative of the circulating *norA* alleles in that hospital at the time (Costa et al., 2011, 2016), which were deposited in GenBank.

A detailed list of all the sequences comprised in this study and respective accession numbers can be found in Supplementary Information S1. The Lisbon MRSA isolates were previously characterized for their susceptibility toward fluoroquinolones and biocides and presence of mutations in the quinolone-resistance determining region of *grlA*/*gyrA* genes (Costa et al., 2011, 2013a) – Supplementary Information S1. Information on the remaining *S. aureus* strains was gathered from the GenBank database and relevant published papers.

Whenever sequence types (ST) were not provided, *in silico* MLST was performed using the MLST 1.8 (Larsen et al., 2012) and SRST2 (Inouye et al., 2014) softwares. The datasets were used to construct a pan-genome (set of all genes within a given species), and a core genome (set of genes found in all strains of that species) for *S. aureus* (Tettelin et al., 2005; van Tonder et al., 2014).

### Sequence Analysis of *norA* and MLST Alleles

*De novo* assemblies were performed for all London samples using Velvet (Zerbino and Birney, 2008) and VelvetOptimiser (Zerbino, 2010). Resulting contig FASTA files and FASTA files of the *S. aureus* samples obtained from GenBank were annotated using Prokka (Seemann, 2014). The pan-genome of these samples was then constructed with Roary (Page et al., 2015), and the sequences of the *norA* and MLST genes (*arcC, aroE, glpF, gmk, pta, tpi*, and *yqiL*) isolated using custom *R* scripts (R Core Team, 2016).

For the set of the nine Portuguese MRSA clinical isolates, *norA* was amplified by PCR using three pairs of primers (Table 1). PCR reaction mixtures were prepared in 0.05 mL containing 2.5 U Taq Polymerase (Thermo Scientific, Waltham, MA, United States); 1X Taq buffer (Thermo Scientific); 30 pmol of each primer (Invitrogen, Carlsbad, CA, United States); 0.2 mM dNTPs (GE Healthcare, Chicago, IL, United States); 1.75 mM MgCl_2_ (Thermo Scientific). The amplification conditions were the following: initial DNA denaturation step at 95°C for 3 min, followed by 35 cycles of denaturation at 94°C for 1 min, annealing at 52°C [norA(a)], 45°C [*norA*(b)] or 50°C [*norA*(c)] for 1 min, and extension at 72°C for 1 min, followed by a final extension step at 72°C for 5 min. The PCR products were sequenced using the same set of primers and the sequences analyzed and assembled to make up the entire fragment using the software MAFFT v. 6^1^ and BioEdit v. 7.0.9.0^2^.

**Table 1:** Primers used to sequence the *norA* promoter and coding region of *Staphylococcus aureus* clinical strains.

Primer	Sequence(5′ – 3′)	Amplicon size	Location^a^	Reference
*norA*_fw(a)	TGTTAAGTCTT GGTCATCTGCA	761	706005–706026	Couto et al., 2008
*norA*_rv(a)	CCATAAATCCA CCAATCCC		706765–706747	This study^b^
*norA*_fw(b)	TTCACCAAGCC ATCAAAAAG	620	706671–706690	Sierra et al., 2000
*norA*_rv(b)	CTTGCCTTTCT CCAGCAATA		707290–707271	Couto et al., 2008
*norA*_fw(c)	GGTCATTATTA TATTCAGTTGTTG	419	707135–707158	Schmitz et al., 1998
*norA*_rv(c)	GTAAGAAAAAC GATGCTAAT		707553–707534	


*^a^Position of the primers in the genome of S. aureus DSM20231 (accession no. CP011526.1). ^b^The primer was designed with the aid of the free software Primer3 (http://primer3.sourceforge.net/) and tested in silico (http://insilico.ehu.es/PCR/).*

The *norA* and MLST gene sequences from all four datasets were aligned using custom *R* scripts. Samples that shared identical *norA* and MLST sequences were grouped together and representative sequences used for phylogenetic reconstruction. Maximum-likelihood (ML) tree construction was performed on the aligned *norA* and concatenated MLST gene sequences separately using RAxML (Stamatakis, 2014) with a GTRGAMMA evolutionary model. The resulting phylogenetic trees were displayed and annotated using FigTree^3^.

### Analysis of the Impact of *norA* Variability on NorA Activity

The tridimensional structure of the NorA efflux pump was predicted via the *in silico* platform PHYRE2 (Protein Homology/analogY Recognition Engine v2.0^4^) (Kelley et al., 2015) based on the nucleotide sequences of the three *norA* variants described in literature. A mutational analysis based on the predicted tridimensional structure was conducted with the Phyre2 Investigator tools, using the SuSPect algorithm (Yates et al., 2014). This algorithm produces a table of scores from 0 to 100 according to predicted deleteriousness (0 = neutral to 100 = deleterious). A score of 50 is recommended as a cut-off between neutral and deleterious variants, with extreme scores being more confident predictions (Yates et al., 2014). In this work, a score of ≥75 was used as cut-off value.

## Results and Discussion

### *norA* Is Part of the Core Genome of *S. aureus*

**FIGURE 1:**
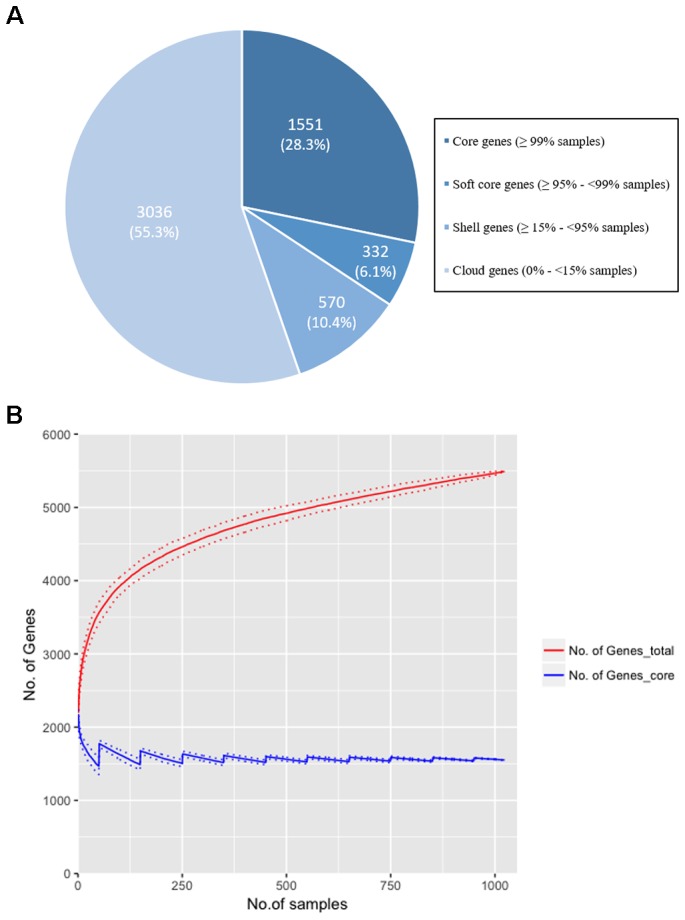
**(A)** The distribution of genes in the pan-genome of the London and GenBank *S. aureus* isolates (*n* = 1029). A total of 5,489 genes were identified with 1,551 (28.25%) genes found in more than 99% of strains. The majority of genes (55.3%) were identified as ‘cloud’ genes, found only in a small number of samples (<15%), demonstrating the diversity of the *S. aureus* genome. **(B)** Rarefaction curves for total (red line) and core (blue line) genes identified when increasing the sample size to reconstruct the pan-genome. Random sampling of samples was conducted 100 times and the mean size of the core genome and number of total genes [and standard deviation (dotted lines)] is shown as the sample size is increased by 1 to the total sample size (*n* = 1029). 90% of the total genes are identified after 514 samples (49.5% of total samples), and there is a plateau in size of the core genome (±5% of the final genome size) at sample 256 (24.9% of total samples). The rarefaction curve confirms that the gene diversity of the strains has been adequately captured with the number of samples included in the analysis.

Our study sought to establish if *norA* is part of the core genome of *S. aureus.* This is a relevant question since a significant part of the studies on efflux-mediated resistance in this pathogen focus mainly on the activity of NorA and some studies have reported the absence of the *norA* gene in several *S. aureus* strains (Monecke and Enricht, 2005; Vali et al., 2008; Conceição et al., 2015; Hasanvand et al., 2015; Liu et al., 2015; Ammar et al., 2016; Taheri et al., 2016; Antiabong et al., 2017; Hassanzadeh et al., 2017; Hadadi et al., 2018; Kernberger-Fischer et al., 2018). This reported absence could be explained by the genetic diversity of the gene and a consequent primer failure during *norA* PCR screening. To test if *norA* is part of the core genome of *S. aureus*, we constructed the pan-genome from the 1029 *S. aureus* isolates whole genome sequence datasets (112 GenBank; 917 London *S. aureus* isolates). The core genome of *S. aureus* consists of 1551 genes (found in ≥99% of individuals with 95% BLAST identity; Figure 1A) with the genome of each individual isolate containing a median of 2196 genes (with a range of 1955 to 2469), marginally lower than the number of genes found in *S. aureus* reference strains (Li et al., 2011). Rarefaction curves were calculated and determined that the number of samples used was adequate to accurately reconstruct the pan-genome of the population; 90% of the total gene diversity was present when considering just 49.5% (514/1029) of the samples and there was a stabilization of the core genome size after 24.9% (256/1029) of the total samples (Figure 1B). The *norA* gene was found to be present in all London and GenBank samples and thus is part of the core genome, with each sample containing a single gene variant. Additionally, we performed a Blastn search using as query the nucleotide sequence of the *norAI* allele (GenBank accession no. D90119.1) against all *S. aureus* genome sequences (complete, scaffold or contig formats) deposited in GenBank^5^, corresponding to more than 8.000 (circa 8.150) sequences. All the Blastn searched sequences retrieved hits with ≥90% identity to *norAI* allele. This result supports the notion that *norA* is a *S. aureus* core gene, occurring in all *S. aureus* genomes. The finding that *norA* is part of *S. aureus* core genome and thus is present in all *S. aureus* strains, implies that reporting the detection of this gene is not sufficient to make a direct association with a particular resistance phenotype. To make such a correlation, one must carry out expression analysis of the *norA* gene as well as of other efflux pump genes, as it has been shown that *S. aureus* strains can display different efflux pump gene expression patterns (DeMarco et al., 2007; Kosmidis et al., 2010, 2012), even under pressure of the same antimicrobial (Huet et al., 2008; Costa et al., 2011, 2015).

### Genetic Diversity of the *norA* Gene

**FIGURE 2:**
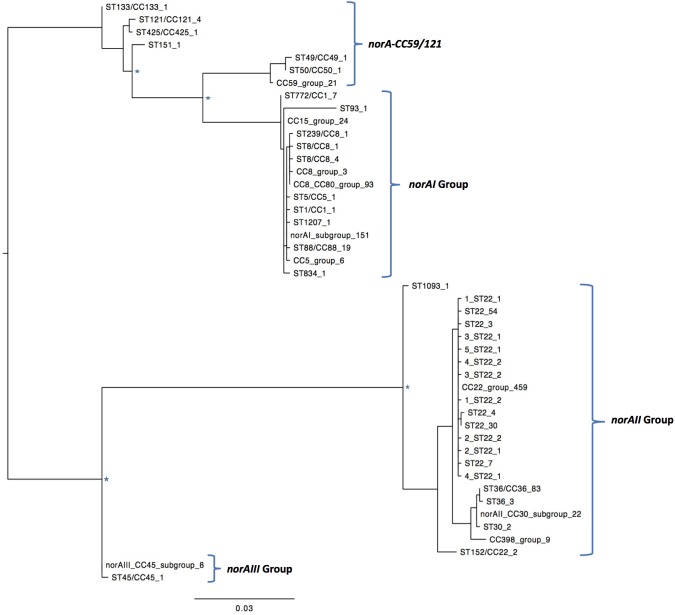
Maximum likelihood phylogeny of the *norA* sequences analyzed in this study. Identical *norA* sequences among all samples used in this study have been collapsed and represented by a single label, which denotes a representative ST/CC of that group. The number of sequences is denoted by the last number of the tip label. Four major groups are found, clustering with the *norA* alleles described in literature, *norAI* (D90119.1), *norAII* (AB019536.1), *norAIII* (M97169.1), and the allelic variant *norA*-CC59/121. The *norAI* associated group is the most diverse, including samples belonging to CC1, CC5, CC8, CC15, CC80, and a wide range of sequence types. The *norAII* associated group contains samples belonging to clonal complexes CC22, CC30, CC36, and CC398. Major nodes supported with bootstrap values above 75% are denoted with a star.

**FIGURE 3:**
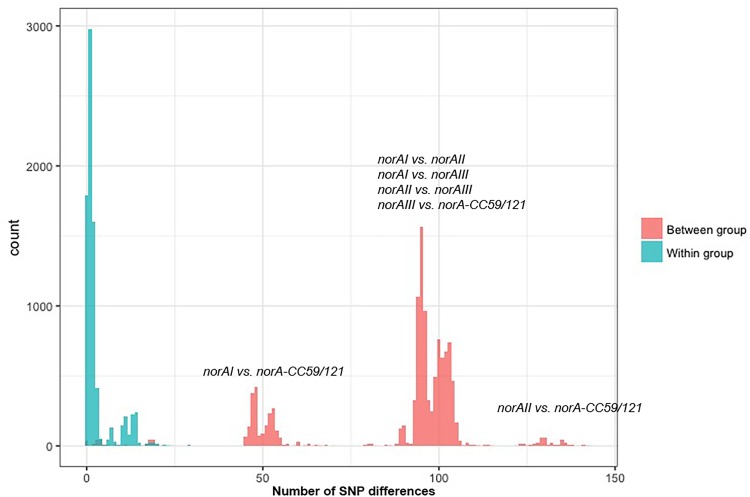
Distribution of the pairwise SNP distance within *norA* sequences used in this study. The *norA* sequences include *norAI* (D90119.1), *norAII* (AB019536.1) and *norAIII* (M97169.1), the nine Portuguese isolates, 112 complete assemblies from GenBank, and 75 representative sequences of London isolates. The figure is annotated to show the pairwise SNP distance between isolates both within (blue) and between (red) each of the four major *norA* groups described in the main text: *norAI* group, *norAII* group, *norAIII* group, and CC121/CC59 group.

One of the goals of this study was to determine the distribution of the *norA* variants amongst the several *S. aureus* clonal lineages. A phylogenetic analysis was performed with the three *norA* alleles and *norA* nucleotide sequences retrieved from complete *S. aureus* genomes available in GenBank and from the Portuguese and London-based *S. aureus* collections (Figure 2). As expected, a certain degree of genetic variability was revealed by the maximum-likelihood tree obtained, despite this gene being relatively conserved. Figure 3 shows the distribution of the pairwise genetic distance within *norA* across the samples used to construct the phylogenetic tree, with a maximum of 143 SNP differences between samples (12.3% of the total gene length of 1164 bp).

A global analysis showed a clear correlation between occurrence of *norA* alleles and specific *S. aureus* clonal lineages. The *norAI* allele was mainly associated with genetic backgrounds of the clonal complexes CC1, CC5, CC8, and CC80 or closely related lineages. On the other hand, *norAII* and close variants appear to be restricted to strains belonging to CC30, CC398, and CC22 (Figure 2). The *norAIII* allele is only associated with the *S. aureus* lineage CC45 and novel but related STs.

Among the sequences retrieved from GenBank, the *norAI* allele is the most frequent (76 out of 112 strains) and the *norAII* allele the second most frequent allele (21 out of 112 strains), whereas *norAIII* occurs in just two CC45 strains. We also detected 13 more divergent variants, most closely related to the *norAI* allele, that were associated with other lineages, such as CC59 and CC121 (Figures 2, 3). Although these findings may be biased as the dataset analyzed reflects the current epidemiologically *S. aureus* relevant clones, with a frequency of ∼70% MRSA strains (78 out of 112 genomes), it clearly shows that *norAI* and related variants are the most prevalent alleles across lineages, as suggested by earlier studies (Schmitz et al., 1998; Sierra et al., 2000; Noguchi et al., 2004). Interestingly, a sample isolated in Brazil in 2010 (FCFHV36) carried a *norAI* type variant of the *norA* gene that included a frameshift mutation in codon 129. This mutation caused a premature stop codon at codon number 147, resulting in potential pseudogenization of *norA* in this isolate.

Regarding the London isolates, we found the *norAII* allele to be the most common allele, identified in 671/917 samples, due to the high number of CC22 strains in the study samples (539/917). As with the GenBank samples, the *norAIII* allele was the least common variant found in the London isolates, with only the three CC45 and four novel ST strains carrying closely related variants. The *norAI* allele was found in 220/917 strains consisting of the broadest range of lineages, comprising 21 different STs. Additionally, one ST22 sample possessed a *norA* variant with a frameshift mutation at codon 365 that results in a premature stop codon at codon number 367 and, again, possible pseudogenization.

**FIGURE 4:**
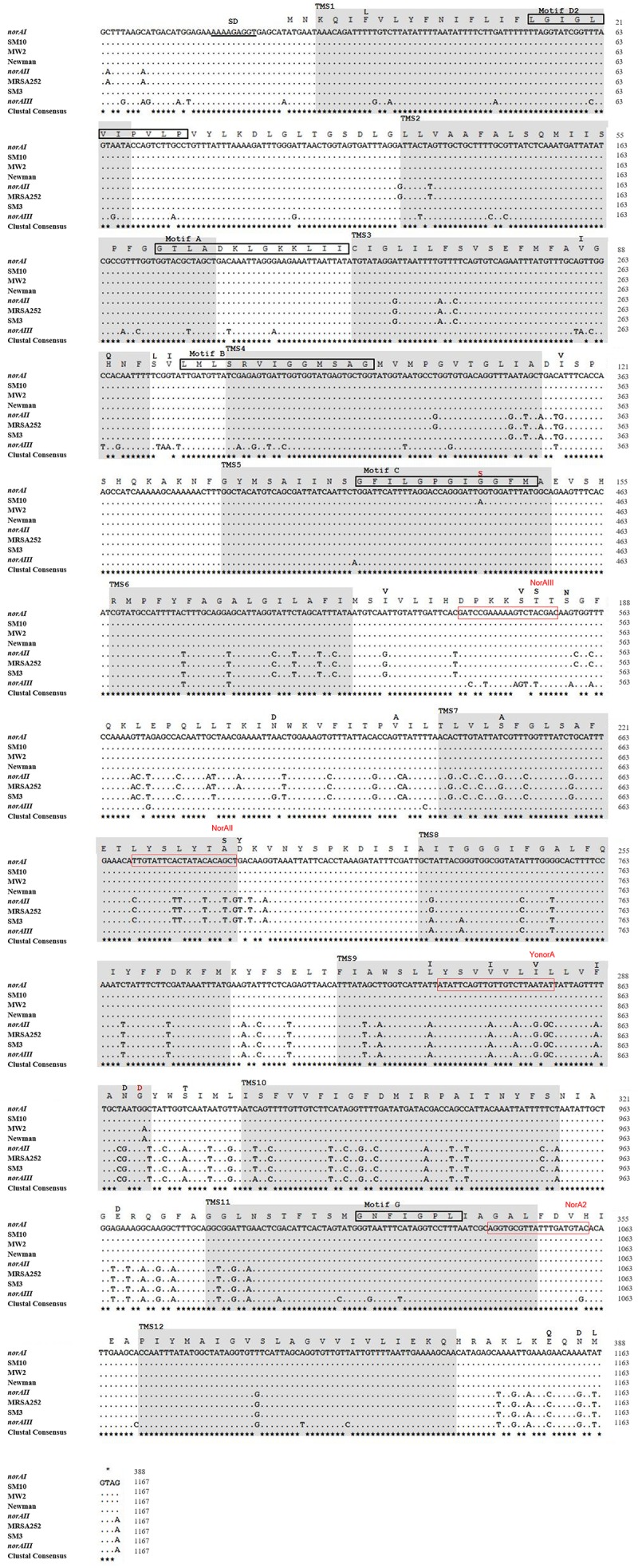
Multiple alignment of *norA* sequences of the three *norA* alleles, from selected Portuguese MRSA clinical isolates (SM) and from strains of representative clonal lineages. The MRSA strains displayed were selected to represent the main clones found, namely SM10 – CC5; SM3 – CC22; MW2 – CC1; Newman – CC8; MRSA252 – CC30. The Shine-Dalgarno (SD) sequence is underlined in black. The shaded regions correspond to the putative TMS of NorA as suggested by Paulsen et al. (1996). The aminoacid regions inside the boxes are relative to the conserved motifs described for MFS transporters with 12 TMS. The black residues shown above the main polypeptide sequence represent the variations present in the various NorA polypeptide sequences relatively to the NorAI variant. The red residues represent alterations found among the SM collection of Portuguese MRSA clinical isolates. The red boxes indicate the primers described in Table 2 for distinction of *norA* alleles.

Nine MRSA isolates, representative of a collection of 53 *S. aureus* clinical isolates isolated at a major Portuguese Hospital and previously characterized for efflux activity and clonal lineage (Costa et al., 2011, 2013a, 2015, 2016) were added to the study to ascertain their *norA* allele. The full sequence of the *norA* gene was determined for these isolates and compared with *norA* alleles from major clonal lineages (Figure 4). Both the *norAI* and *norAII* alleles were found amongst the Portuguese isolates and their distribution reflects the findings for the other datasets analyzed. Eight isolates from clonal lineages CC5, CC8, and CC88 carried *norAI* and a single isolate from clonal lineage CC22 harboured the *norAII* allele (Figure 4). Combining information on *norA* allele, clonal lineage and previous characterization of these isolates (Costa et al., 2011) showed no correlation between *norA* allele, *norA* expression levels, efflux capacity, or antimicrobial resistance levels (Supplementary Information S1).

A wet lab PCR-based strategic approach is proposed for detection and differentiation of the several *norA* alleles occurring in *S. aureus*. The *norAI* and *norAII* alleles can be easily distinguished using two sets of specific primers (Table 2 and Figure 4). To amplify and differentiate *norAIII* from the *norA* allele associated with CC59/CC121, an approach consisting in PCR amplification followed by restriction of the PCR product with HindIII can be applied (Table 2 and Figure 4).

**Table 2:** List of primers proposed to amplify and differentiate the *norA* alleles by conventional PCR.

Primer	Sequence(5′ – 3′)	Amplified *norA* allele	Amplicon size (nt)	Reference
*norA*_fw(b)	TTCACCAAGC CATCAAAAAG	all	704	Sierra et al., 2000
NorA2	GCACATCAAA TAACGCACCT			
Yo*norA*	ATATTCAGTTGT TGTCTTAATAT	*norAI*	230	Sierra et al., 2000
NorA2	GCACATCAAA TAACGCACCT			
NorAII	CTGTATTCTTT ATATACATCG	*norAII*	391	This study^a^
NorA2	GCACATCAAAT AACGCACCT			Sierra et al., 2000
NorAIII	GACCCTAAAA AAGTTTCGAC	*norAIII*	526	This study^a^
NorA2	GCACATCAAATA ACGCACCT	*norA-CC59/CC121*		Sierra et al., 2000
	Plus HindIII restriction:			
	*norAIII*: 166 nt + 360 nt;			
	*norA-CC59/121*: no cut (526 nt)			


*^a^The primers were designed with the aid of the free software Primer3 (http://primer3.sourceforge.net/). The primers and the HindIII restriction were tested in silico (http://insilico.ehu.eus/) against several S. aureus genome sequences of representative clonal lineages.*

### Impact of Genetic Variability on NorA Function

A mutational analysis was performed *in silico* via the PHYRE2 platform to construct a NorA structural model, using the SuSPect algorithm to predict the effect of each residue substitution on NorA function. This analysis is limited because of the lack of available structural data for bacterial MFS pumps, mostly limited to substrate-specific transporters. Nevertheless, the polypeptide sequences encoded by the three *norAI* alleles were used to predict a tri-dimensional structure of NorA. All three NorA variants showed the highest identity with the putative MFS transporter YajR of *Escherichia coli* (Jiang et al., 2013). A second NorA *in silico* model, based on the structure of glycerol-3-phosphate MFS transporter from *E. coli*, described by Bhaskar et al. (2016) was taken into account for comparison purposes. The SuSPect-derived mutational analysis revealed 42 residues for which particular mutations could potentially impair NorA activity (Supplementary Information S2). Of these, residues Pro110, Pro158, Pro311, and Gly326 were particularly susceptible to mutations.

**FIGURE 5:**
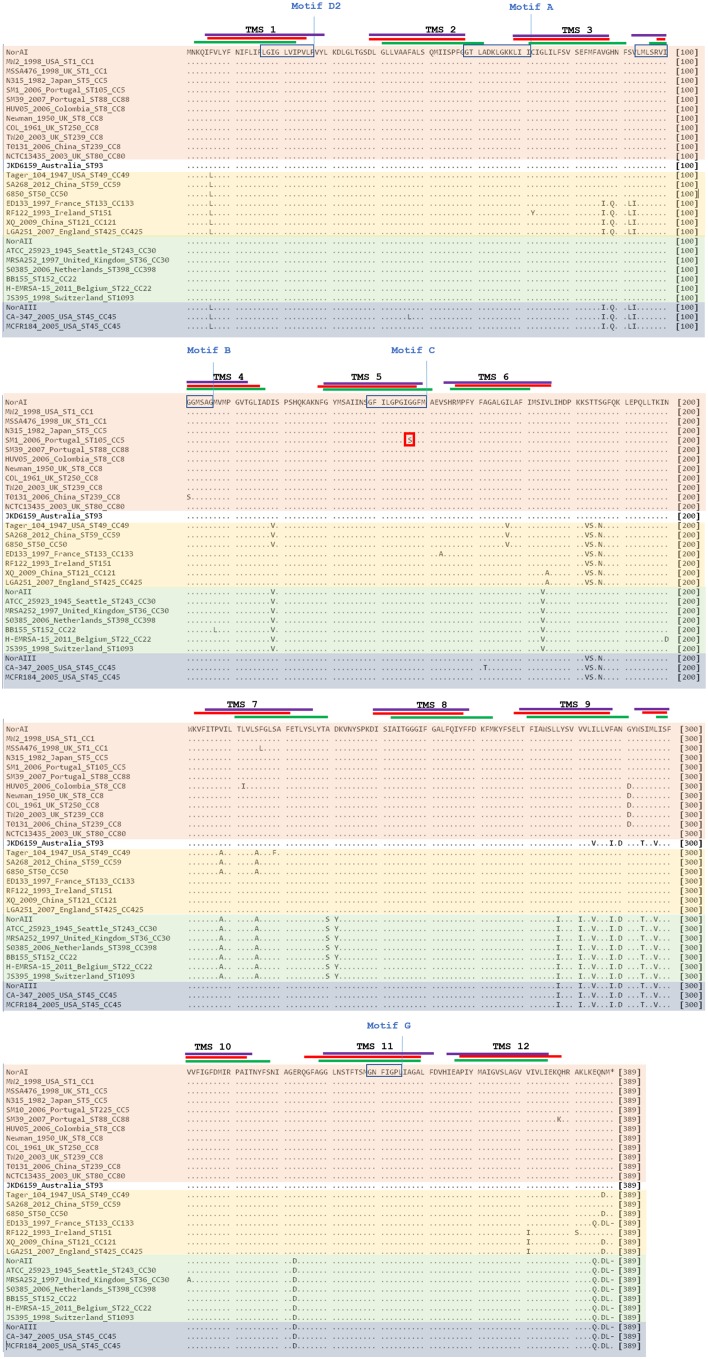
Multiple alignment of NorA polypeptide sequences derived from alleles *norAI* (Yoshida et al., 1990), *norAII* (Noguchi et al., 2004), and *norAIII* (Kaatz et al., 1993) and from the *norA* alleles from selected *S. aureus* strains. The MRSA strains displayed were selected to represent the main *S. aureus* clonal lineages found for the strains with genomes publicly available and for the Portuguese strains (SM10 – CC5; SM39 – CC88). Sequences are grouped according to the respective *norA* allele (orange – *norAI*; green – *norAII*; blue - *norAIII*; yellow – *norA-CC59/121*). The lines correspond to the transmembrane segments (TMS) of NorA, as predicted by Paulsen et al. (1996) (green), by the HMMTOP method as presented in Bhaskhar et al. (2016) (red) and as predicted by the Phyre2-based NorA model (purple). The aminoacid regions inside the boxes are relative to the conserved motifs described for MFS transporters with 12 TMS (Paulsen et al., 1996). The red box corresponds to the amino acid substitution likely to affect NorA function.

Analysis of the polypeptide sequence corresponding to the three *norA* variants revealed a total of 43 amino acid alterations. Figure 5 summarizes these alterations, their location in NorA and their distribution amongst *S. aureus* lineages. There was no overlap between these alterations and the residues identified by SuSPect. We also compared the *S. aureus* NorAI variant with the NorA efflux pump encoded in the *Staphylococcus epidermidis* ATCC 12228 chromosome, which presents the same substrate profile (Costa et al., 2018; Supplementary Information S2). The differences encountered between the two polypeptide sequences correspond to residues that were not highlighted as pivotal for the protein activity by SuSPect. These results suggest conservation of NorA function in these two main staphylococcal species.

One alteration, Gly147Ser located within putative TMS 5, may affect NorA function (Figure 4). This alteration was detected in all four strains belonging to ST105 (three of which are of Portuguese origin) and in one of the strains belonging to ST225 (Portuguese origin) (Supplementary Information S2), but was absent from strains from other CC5 lineages, such as ST5. Residue 147 is predicted to be located within the conserved motif C (gxxxGPxiGGxl) (Paulsen et al., 1996) on the TMS 5, facing a water-filled channel. This motif is conserved among MFS drug-H^+^ antiporters and has been implicated in the binding of the substrate, with the glycines being essential for that function (Ginn et al., 2000; Tamura et al., 2001). In fact, in a mutagenesis study in *S. aureus* efflux pump TetA(K), also a 12-TMS member of the MFS family, the mutation Gly147Ser was responsible for the loss of 80% of the activity of that efflux pump (Ginn et al., 2000). Although Tet transporters and NorA are distinct in their substrate specificity, these pumps share abundance of glycines in TMS 5, a trait that has been postulated to confer conformational plasticity to EPs (Ginn et al., 2000). Thus, the mutation Gly147Ser may have a deleterious effect on NorA activity. Additional studies are necessary to fully ascertain the impact of this alteration in the efflux capacity of NorA. The same applies to the full understanding of possible correlations between a given *norA* allele and corresponding NorA efflux capacity, probably requiring carefully controlled genetic systems because of overlapping substrates between many staphylococcal multidrug efflux systems and the redundancy in their response to antimicrobials (Costa et al., 2013b; Schindler and Kaatz, 2016).

## Conclusion

In this study we provide evidence that the efflux pump gene *norA* is part of core genome of *S. aureus*. This gene presents some genetic variability which may impair its detection by conventional laboratorial techniques, such as PCR, due to potential primer mismatch. Strikingly, a correlation was observed between the several *norA* alleles and specific *S. aureus* lineages, suggesting that the occurrence of *norA* variants reflects the population structure of this major pathogen.

## Data Availability

The sequences analyzed in this study are available in GenBank and their accession numbers detailed in Supplementary Information S1, except for WGS data for the London-based strains that are available from the European Nucleotide Archive database under accession number PRJEB11177.

## Author Contributions

IC, SC, and TC conceived and designed the study. SC performed the wet lab *norA* analysis and the *in silico* mutational analysis. BS conducted the pan-genome analysis. SC and BS performed the phylogenetic analysis. JE provided genomic data on the London-based MRSA strains. RP, JE, MV, TC, and IC contributed to the analysis of these data. SC, BS, TC, and IC wrote the first draft of the manuscript. All authors have reviewed the final version of the manuscript.

## Conflict of Interest Statement

The authors declare that the research was conducted in the absence of any commercial or financial relationships that could be construed as a potential conflict of interest.

## Footnotes

1 mafft.cbrc.jp/alignment/software/

2 https://bioedit.software.informer.com/

3 http://tree.bio.ed.ac.uk/software/figtree/

4 http://www.sbg.bio.ic.ac.uk/phyre2

5 www.ncbi.nlm.nih.gov/genome/genomes/154
