# Supplementary material for: Genetic Diversity of norA, Coding for a Main Efflux Pump of Staphylococcus aureus
Source: Front Genet. 2019 Jan 9;9:710. doi: 10.3389/fgene.2018.00710 (PMC6333699; doi:10.3389/fgene.2018.00710)
Supplement: Supplementary file 2 [file Data_Sheet_2.docx]

Supplementary Material

Genetic diversity of *norA*, coding for a main efflux pump of *Staphylococcus aureus*

Sofia Santos Costa^1^, Benjamin Sobkowiak^2^, Ricardo Parreira^1^, Jonathan D. Edgeworth^3^, Miguel Viveiros^1^, Taane G. Clark^2,4^**, Isabel Couto^1^*

***Correspondence:** Isabel Couto: [icouto@ihmt.un.pt](mailto:icouto@ihmt.un.pt);

***Co-corresponding author:** Taane G. Clark: taane.clark@lshtm.ac.uk

**Table ST1. *S. aureus* (*Sau*) NorA mutational analysis based on SuSPect algorithm available at the PHYRE2 plattform.** The effect on NorA activity of each possible mutation is given as a score (0🡪100) and color (blue🡪red) code, where 0/blue represent no effect and 100/red represents a strong deleterious impact on protein activity. NorA residues shadowed in light red correspond to the ones for which at least one alteration promotes a high impact on protein activity (score ≥ 75). A comparison with *S. epidermidis* (*Sep*) NorA sequence is also provided (* consensus).

| ***Sau* NorAI** | | **Alternative aminoacid** | | | | | | | | | | | | | | | | | | | | ***Sep***  **NorA** |
| --- | --- | --- | --- | --- | --- | --- | --- | --- | --- | --- | --- | --- | --- | --- | --- | --- | --- | --- | --- | --- | --- | --- |
|  |  | **A** | **C** | **D** | **E** | **F** | **G** | **H** | **I** | **K** | **L** | **M** | **N** | **P** | **Q** | **R** | **S** | **T** | **V** | **W** | **Y** |  |
| **1** | **M** | 35 | 39 | 44 | 37 | 34 | 50 | 41 | 19 | 43 | 14 | 3 | 40 | 30 | 35 | 43 | 38 | 36 | 21 | 40 | 36 | ***** |
| **2** | **N** | 18 | 21 | 12 | 15 | 18 | 28 | 10 | 20 | 5 | 19 | 18 | 2 | 10 | 12 | 9 | 11 | 10 | 22 | 23 | 20 | **K** |
| **3** | **K** | 27 | 46 | 52 | 30 | 40 | 52 | 25 | 36 | 2 | 39 | 35 | 25 | 19 | 22 | 7 | 28 | 27 | 46 | 46 | 34 | ***** |
| **4** | **Q** | 11 | 27 | 15 | 8 | 25 | 52 | 15 | 19 | 19 | 22 | 14 | 11 | 10 | 2 | 17 | 11 | 11 | 20 | 26 | 22 | ***** |
| **5** | **I** | 24 | 36 | 57 | 51 | 12 | 79 | 46 | 2 | 51 | 4 | 13 | 55 | 27 | 46 | 60 | 33 | 29 | 7 | 43 | 26 | **L** |
| **6** | **F** | 23 | 43 | 61 | 60 | 2 | 59 | 44 | 15 | 54 | 12 | 23 | 54 | 30 | 45 | 46 | 32 | 28 | 21 | 7 | 8 | ***** |
| **7** | **V** | 13 | 23 | 49 | 42 | 19 | 61 | 46 | 6 | 50 | 10 | 11 | 47 | 11 | 42 | 59 | 23 | 7 | 2 | 28 | 31 | **I** |
| **8** | **L** | 42 | 34 | 77 | 72 | 29 | 88 | 71 | 12 | 81 | 2 | 16 | 72 | 41 | 68 | 72 | 60 | 41 | 18 | 61 | 52 | ***** |
| **9** | **Y** | 14 | 18 | 51 | 43 | 5 | 62 | 14 | 16 | 50 | 12 | 12 | 29 | 29 | 30 | 47 | 21 | 25 | 17 | 6 | 1 | ***** |
| **10** | **F** | 19 | 33 | 56 | 52 | 2 | 55 | 42 | 12 | 64 | 11 | 17 | 52 | 32 | 54 | 63 | 25 | 28 | 16 | 21 | 17 | ***** |
| **11** | **N** | 10 | 12 | 19 | 21 | 25 | 14 | 15 | 13 | 26 | 16 | 13 | 1 | 16 | 20 | 27 | 6 | 8 | 14 | 36 | 25 | ***** |
| **12** | **I** | 14 | 17 | 43 | 31 | 16 | 55 | 43 | 2 | 45 | 8 | 7 | 18 | 17 | 27 | 47 | 22 | 13 | 5 | 31 | 22 | ***** |
| **13** | **F** | 32 | 43 | 71 | 68 | 1 | 79 | 60 | 36 | 78 | 19 | 27 | 57 | 48 | 71 | 77 | 38 | 47 | 30 | 41 | 27 | ***** |
| **14** | **L** | 26 | 24 | 54 | 60 | 15 | 77 | 59 | 7 | 70 | 2 | 8 | 58 | 32 | 55 | 65 | 34 | 24 | 11 | 47 | 42 | ***** |
| **15** | **I** | 15 | 21 | 33 | 39 | 17 | 55 | 34 | 2 | 60 | 11 | 13 | 29 | 23 | 45 | 40 | 23 | 19 | 7 | 48 | 28 | ***** |
| **16** | **F** | 20 | 25 | 51 | 51 | 1 | 48 | 31 | 18 | 60 | 16 | 11 | 35 | 23 | 39 | 43 | 27 | 23 | 18 | 13 | 9 | ***** |
| **17** | **L** | 17 | 17 | 50 | 45 | 11 | 64 | 43 | 7 | 56 | 2 | 6 | 44 | 20 | 38 | 49 | 23 | 13 | 5 | 34 | 30 | ***** |
| **18** | **G** | 20 | 43 | 25 | 52 | 50 | 1 | 53 | 54 | 62 | 60 | 51 | 35 | 34 | 51 | 62 | 16 | 37 | 47 | 56 | 61 | ***** |

Table ST1. *Cont.*

| ***Sau* NorAI** | | **Alternative aminoacid** | | | | | | | | | | | | | | | | | | | | ***Sep***  **NorA** |
| --- | --- | --- | --- | --- | --- | --- | --- | --- | --- | --- | --- | --- | --- | --- | --- | --- | --- | --- | --- | --- | --- | --- |
|  |  | **A** | **C** | **D** | **E** | **F** | **G** | **H** | **I** | **K** | **L** | **M** | **N** | **P** | **Q** | **R** | **S** | **T** | **V** | **W** | **Y** |  |
| **19** | **I** | 15 | 27 | 41 | 27 | 8 | 55 | 37 | 2 | 50 | 9 | 8 | 24 | 19 | 20 | 36 | 19 | 17 | 6 | 26 | 13 | ***** |
| **20** | **G** | 18 | 39 | 29 | 41 | 21 | 2 | 30 | 43 | 47 | 41 | 27 | 19 | 21 | 33 | 46 | 11 | 19 | 40 | 21 | 25 | ***** |
| **21** | **L** | 23 | 26 | 55 | 39 | 15 | 72 | 48 | 5 | 60 | 1 | 4 | 50 | 24 | 39 | 55 | 27 | 26 | 8 | 40 | 24 | ***** |
| **22** | **V** | 27 | 30 | 64 | 59 | 33 | 73 | 57 | 6 | 71 | 16 | 18 | 65 | 25 | 42 | 73 | 43 | 27 | 2 | 54 | 32 | ***** |
| **23** | **I** | 19 | 37 | 47 | 53 | 16 | 71 | 42 | 2 | 65 | 9 | 10 | 33 | 26 | 49 | 64 | 24 | 24 | 10 | 33 | 28 | ***** |
| **24** | **P** | 46 | 76 | 69 | 65 | 72 | 75 | 73 | 66 | 79 | 73 | 65 | 73 | 1 | 71 | 83 | 58 | 47 | 59 | 79 | 74 | ***** |
| **25** | **V** | 14 | 35 | 53 | 37 | 19 | 76 | 52 | 5 | 61 | 10 | 15 | 49 | 23 | 44 | 67 | 35 | 19 | 2 | 44 | 30 | ***** |
| **26** | **L** | 34 | 43 | 64 | 41 | 17 | 81 | 44 | 13 | 71 | 1 | 7 | 53 | 32 | 49 | 66 | 47 | 23 | 20 | 46 | 42 | ***** |
| **27** | **P** | 44 | 78 | 66 | 66 | 79 | 78 | 74 | 73 | 78 | 77 | 64 | 70 | 1 | 61 | 83 | 43 | 50 | 60 | 82 | 68 | ***** |
| **28** | **V** | 10 | 19 | 22 | 17 | 12 | 54 | 25 | 4 | 26 | 7 | 9 | 25 | 7 | 21 | 36 | 13 | 9 | 1 | 23 | 13 | ***** |
| **29** | **Y** | 45 | 63 | 69 | 68 | 10 | 89 | 30 | 20 | 70 | 22 | 25 | 68 | 53 | 49 | 69 | 65 | 52 | 35 | 35 | 1 | ***** |
| **30** | **L** | 11 | 28 | 56 | 45 | 18 | 65 | 47 | 6 | 39 | 1 | 6 | 50 | 22 | 24 | 39 | 18 | 21 | 9 | 40 | 32 | ***** |
| **31** | **K** | 6 | 13 | 10 | 5 | 11 | 27 | 6 | 11 | 1 | 10 | 7 | 5 | 6 | 5 | 3 | 7 | 6 | 10 | 13 | 9 | ***** |
| **32** | **D** | 14 | 28 | 1 | 5 | 29 | 53 | 12 | 21 | 29 | 30 | 23 | 12 | 11 | 11 | 35 | 8 | 16 | 19 | 32 | 20 | ***** |
| **33** | **L** | 34 | 44 | 65 | 47 | 12 | 84 | 56 | 15 | 59 | 1 | 11 | 60 | 27 | 39 | 53 | 46 | 43 | 21 | 30 | 20 | ***** |
| **34** | **G** | 6 | 12 | 7 | 8 | 12 | 1 | 4 | 10 | 9 | 12 | 8 | 5 | 6 | 7 | 11 | 6 | 9 | 11 | 10 | 12 | ***** |
| **35** | **L** | 14 | 29 | 36 | 37 | 18 | 50 | 34 | 9 | 45 | 2 | 13 | 35 | 19 | 26 | 42 | 22 | 21 | 12 | 25 | 21 | ***** |
| **36** | **T** | 10 | 18 | 12 | 14 | 21 | 22 | 14 | 18 | 7 | 16 | 14 | 9 | 7 | 14 | 15 | 6 | 2 | 14 | 21 | 21 | **K** |
| **37** | **G** | 6 | 20 | 13 | 13 | 19 | 2 | 15 | 20 | 19 | 19 | 16 | 9 | 5 | 17 | 23 | 7 | 11 | 16 | 19 | 17 | ***** |
| **38** | **S** | 6 | 18 | 12 | 11 | 10 | 26 | 13 | 16 | 24 | 15 | 12 | 11 | 8 | 10 | 26 | 1 | 7 | 13 | 15 | 18 | ***** |
| **39** | **D** | 13 | 28 | 1 | 5 | 21 | 49 | 12 | 16 | 37 | 18 | 14 | 16 | 12 | 6 | 47 | 11 | 12 | 15 | 15 | 25 | ***** |
| **40** | **L** | 8 | 20 | 45 | 31 | 10 | 56 | 37 | 4 | 42 | 1 | 5 | 28 | 14 | 31 | 43 | 18 | 14 | 5 | 24 | 15 | ***** |
| **41** | **G** | 24 | 58 | 54 | 46 | 55 | 1 | 54 | 62 | 61 | 64 | 54 | 28 | 32 | 35 | 61 | 19 | 35 | 57 | 55 | 62 | ***** |
| **42** | **L** | 17 | 27 | 45 | 39 | 12 | 65 | 31 | 3 | 53 | 1 | 6 | 36 | 14 | 26 | 42 | 26 | 19 | 10 | 8 | 12 | **M** |
| **43** | **L** | 19 | 32 | 57 | 54 | 18 | 80 | 50 | 8 | 62 | 1 | 9 | 51 | 22 | 49 | 59 | 25 | 22 | 10 | 37 | 36 | ***** |
| **44** | **V** | 13 | 28 | 48 | 34 | 13 | 57 | 46 | 5 | 59 | 9 | 8 | 36 | 20 | 33 | 63 | 19 | 14 | 1 | 43 | 23 | ***** |
| **45** | **A** | 1 | 23 | 39 | 37 | 30 | 22 | 42 | 25 | 51 | 26 | 20 | 32 | 12 | 39 | 53 | 6 | 9 | 20 | 44 | 40 | ***** |
| **46** | **A** | 1 | 8 | 31 | 28 | 16 | 30 | 29 | 8 | 40 | 11 | 14 | 30 | 9 | 29 | 40 | 8 | 10 | 3 | 31 | 26 | ***** |
| **47** | **F** | 24 | 34 | 49 | 37 | 1 | 67 | 28 | 29 | 61 | 24 | 16 | 25 | 17 | 40 | 54 | 17 | 27 | 31 | 17 | 3 | ***** |
| **48** | **A** | 1 | 21 | 39 | 34 | 19 | 27 | 39 | 26 | 48 | 19 | 14 | 25 | 8 | 34 | 47 | 7 | 13 | 20 | 36 | 23 | ***** |
| **49** | **L** | 16 | 27 | 55 | 51 | 10 | 60 | 50 | 6 | 61 | 1 | 12 | 52 | 25 | 48 | 56 | 36 | 26 | 10 | 31 | 31 | ***** |
| **50** | **S** | 5 | 10 | 22 | 21 | 16 | 24 | 25 | 15 | 37 | 17 | 8 | 18 | 8 | 24 | 43 | 1 | 5 | 12 | 34 | 30 | ***** |
| **51** | **Q** | 19 | 45 | 32 | 17 | 32 | 59 | 27 | 30 | 33 | 29 | 14 | 24 | 21 | 1 | 26 | 15 | 24 | 25 | 21 | 19 | ***** |
| **52** | **M** | 11 | 16 | 45 | 40 | 8 | 45 | 38 | 10 | 45 | 6 | 1 | 37 | 16 | 20 | 43 | 17 | 14 | 9 | 33 | 30 | ***** |

**Table ST1. *Cont.***

| ***Sau* NorAI** | | | **Alternative aminoacid** | | | | | | | | | | | | | | | | | | | | | ***Sep***  **NorA** |
| --- | --- | --- | --- | --- | --- | --- | --- | --- | --- | --- | --- | --- | --- | --- | --- | --- | --- | --- | --- | --- | --- | --- | --- | --- |
|  |  |  | **A** | | **C** | **D** | **E** | **F** | **G** | **H** | **I** | **K** | **L** | **M** | **N** | **P** | **Q** | **R** | **S** | **T** | **V** | **W** | **Y** |  |
| **53** | **I** | 16 | | 17 | | 49 | 46 | 10 | 61 | 45 | 1 | 58 | 6 | 17 | 47 | 17 | 48 | 56 | 30 | 20 | 5 | 39 | 29 | ***** |
| **54** | **I** | 7 | | 7 | | 32 | 32 | 4 | 27 | 30 | 1 | 41 | 5 | 5 | 30 | 11 | 32 | 40 | 9 | 8 | 4 | 26 | 18 | ***** |
| **55** | **S** | 8 | | 23 | | 30 | 21 | 39 | 42 | 34 | 31 | 34 | 30 | 16 | 21 | 20 | 13 | 29 | 2 | 13 | 28 | 46 | 41 | ***** |
| **56** | **P** | 44 | | 72 | | 67 | 63 | 52 | 88 | 69 | 43 | 74 | 41 | 50 | 71 | 1 | 67 | 79 | 62 | 54 | 55 | 66 | 65 | ***** |
| **57** | **F** | 17 | | 29 | | 45 | 42 | 1 | 57 | 34 | 6 | 48 | 8 | 14 | 42 | 9 | 35 | 46 | 21 | 22 | 11 | 12 | 7 | ***** |
| **58** | **G** | 7 | | 18 | | 31 | 33 | 15 | 2 | 22 | 17 | 38 | 18 | 14 | 21 | 16 | 27 | 37 | 11 | 17 | 15 | 6 | 18 | ***** |
| **59** | **G** | 28 | | 65 | | 57 | 60 | 65 | 1 | 62 | 66 | 69 | 67 | 61 | 46 | 43 | 63 | 68 | 26 | 47 | 61 | 62 | 65 | ***** |
| **60** | **T** | 5 | | 13 | | 16 | 12 | 11 | 33 | 11 | 9 | 11 | 9 | 8 | 8 | 4 | 12 | 7 | 5 | 1 | 7 | 7 | 10 | ***** |
| **61** | **L** | 24 | | 25 | | 49 | 46 | 14 | 66 | 43 | 9 | 43 | 1 | 5 | 43 | 18 | 43 | 40 | 35 | 32 | 16 | 12 | 21 | ***** |
| **62** | **A** | 1 | | 20 | | 47 | 44 | 36 | 31 | 44 | 26 | 57 | 27 | 34 | 45 | 18 | 45 | 59 | 6 | 15 | 17 | 53 | 41 | ***** |
| **63** | **D** | 34 | | 54 | | 0 | 14 | 56 | 50 | 38 | 54 | 56 | 52 | 53 | 28 | 29 | 38 | 61 | 33 | 41 | 51 | 56 | 54 | ***** |
| **64** | **K** | 11 | | 19 | | 26 | 14 | 24 | 37 | 10 | 16 | 1 | 16 | 14 | 11 | 14 | 8 | 3 | 9 | 11 | 18 | 23 | 17 | ***** |
| **65** | **L** | 17 | | 16 | | 38 | 26 | 5 | 52 | 18 | 5 | 25 | 1 | 7 | 25 | 18 | 22 | 21 | 18 | 17 | 7 | 9 | 7 | ***** |
| **66** | **G** | 9 | | 24 | | 12 | 17 | 26 | 0 | 16 | 28 | 20 | 27 | 22 | 8 | 8 | 18 | 19 | 10 | 19 | 27 | 21 | 22 | ***** |
| **67** | **K** | 16 | | 20 | | 25 | 14 | 19 | 30 | 13 | 25 | 1 | 25 | 18 | 13 | 8 | 10 | 2 | 11 | 12 | 23 | 22 | 15 | ***** |
| **68** | **K** | 27 | | 38 | | 39 | 22 | 32 | 47 | 22 | 32 | 1 | 35 | 27 | 22 | 17 | 19 | 4 | 26 | 25 | 23 | 27 | 23 | ***** |
| **69** | **L** | 11 | | 14 | | 29 | 20 | 9 | 54 | 15 | 4 | 10 | 1 | 6 | 11 | 2 | 13 | 8 | 10 | 8 | 8 | 8 | 12 | ***** |
| **70** | **I** | 26 | | 38 | | 65 | 64 | 20 | 82 | 62 | 1 | 74 | 8 | 9 | 63 | 15 | 50 | 70 | 38 | 21 | 5 | 42 | 38 | ***** |
| **71** | **I** | 38 | | 42 | | 71 | 69 | 19 | 88 | 66 | 1 | 79 | 6 | 11 | 68 | 40 | 63 | 77 | 56 | 41 | 14 | 60 | 30 | ***** |
| **72** | **C** | 5 | | 0 | | 30 | 21 | 8 | 51 | 26 | 4 | 37 | 4 | 6 | 25 | 12 | 21 | 26 | 9 | 9 | 4 | 18 | 15 | ***** |
| **73** | **I** | 14 | | 24 | | 54 | 54 | 12 | 66 | 52 | 2 | 63 | 8 | 15 | 53 | 15 | 52 | 46 | 22 | 22 | 6 | 22 | 22 | ***** |
| **74** | **G** | 25 | | 33 | | 60 | 66 | 63 | 1 | 64 | 67 | 73 | 59 | 57 | 51 | 44 | 67 | 73 | 19 | 43 | 66 | 65 | 69 | ***** |
| **75** | **L** | 32 | | 27 | | 69 | 62 | 22 | 76 | 59 | 10 | 72 | 2 | 8 | 52 | 34 | 46 | 69 | 40 | 29 | 16 | 52 | 36 | ***** |
| **76** | **I** | 9 | | 14 | | 39 | 37 | 8 | 43 | 35 | 1 | 47 | 5 | 12 | 35 | 14 | 37 | 46 | 15 | 14 | 3 | 33 | 19 | **V** |
| **77** | **L** | 16 | | 16 | | 53 | 49 | 13 | 43 | 47 | 6 | 58 | 1 | 9 | 47 | 20 | 44 | 55 | 23 | 20 | 8 | 32 | 32 | **F** |
| **78** | **F** | 38 | | 40 | | 74 | 59 | 1 | 76 | 60 | 39 | 83 | 27 | 26 | 56 | 57 | 69 | 78 | 37 | 34 | 35 | 23 | 10 | ***** |
| **79** | **S** | 2 | | 10 | | 18 | 16 | 14 | 26 | 20 | 11 | 31 | 12 | 9 | 14 | 11 | 20 | 36 | 1 | 6 | 14 | 27 | 20 | **A** |
| **80** | **V** | 9 | | 13 | | 40 | 35 | 12 | 56 | 38 | 2 | 51 | 6 | 13 | 40 | 12 | 37 | 54 | 18 | 15 | 1 | 35 | 22 | ***** |
| **81** | **S** | 7 | | 20 | | 21 | 29 | 20 | 26 | 35 | 23 | 48 | 26 | 17 | 23 | 19 | 32 | 55 | 1 | 13 | 24 | 45 | 34 | ***** |
| **82** | **E** | 9 | | 18 | | 6 | 1 | 10 | 30 | 11 | 19 | 23 | 12 | 10 | 6 | 5 | 5 | 29 | 4 | 9 | 14 | 14 | 9 | ***** |
| **83** | **F** | 15 | | 26 | | 39 | 48 | 1 | 48 | 38 | 10 | 58 | 7 | 15 | 48 | 25 | 47 | 59 | 21 | 23 | 10 | 12 | 12 | ***** |
| **84** | **M** | 8 | | 13 | | 34 | 32 | 10 | 30 | 31 | 6 | 38 | 2 | 1 | 27 | 11 | 15 | 36 | 13 | 15 | 6 | 17 | 22 | ***** |
| **85** | **F** | 19 | | 11 | | 56 | 56 | 1 | 65 | 37 | 19 | 65 | 13 | 14 | 53 | 23 | 40 | 67 | 21 | 21 | 23 | 15 | 6 | ***** |
| **86** | **A** | 1 | | 25 | | 51 | 43 | 25 | 18 | 41 | 25 | 58 | 23 | 24 | 32 | 10 | 35 | 52 | 15 | 21 | 20 | 28 | 24 | ***** |
| **87** | **V** | 9 | | 9 | | 30 | 18 | 5 | 51 | 24 | 2 | 36 | 4 | 6 | 20 | 7 | 15 | 34 | 14 | 9 | 1 | 11 | 8 | **A** |

**Table ST1. *Continuation.***

| ***Sau* NorAI** | | **Alternative aminoacid** | | | | | | | | | | | | | | | | | | | | ***Sep***  **NorA** |
| --- | --- | --- | --- | --- | --- | --- | --- | --- | --- | --- | --- | --- | --- | --- | --- | --- | --- | --- | --- | --- | --- | --- |
|  |  | **A** | **C** | **D** | **E** | **F** | **G** | **H** | **I** | **K** | **L** | **M** | **N** | **P** | **Q** | **R** | **S** | **T** | **V** | **W** | **Y** |  |
| **88** | **G** | 3 | 14 | 23 | 20 | 14 | 2 | 19 | 16 | 30 | 17 | 18 | 13 | 10 | 18 | 25 | 4 | 11 | 11 | 10 | 19 | ***** |
| **89** | **H** | 16 | 27 | 10 | 11 | 21 | 37 | 1 | 23 | 20 | 20 | 22 | 7 | 6 | 8 | 17 | 10 | 10 | 19 | 13 | 10 | **Q** |
| **90** | **N** | 18 | 34 | 11 | 21 | 31 | 33 | 5 | 31 | 26 | 30 | 27 | 1 | 17 | 17 | 27 | 7 | 13 | 30 | 41 | 33 | **S** |
| **91** | **F** | 21 | 41 | 49 | 47 | 1 | 67 | 36 | 11 | 46 | 11 | 13 | 53 | 10 | 52 | 61 | 37 | 30 | 14 | 15 | 6 | ***** |
| **92** | **S** | 4 | 9 | 9 | 5 | 7 | 19 | 7 | 7 | 14 | 8 | 7 | 6 | 1 | 8 | 21 | 1 | 4 | 6 | 4 | 7 | **T** |
| **93** | **V** | 11 | 17 | 29 | 18 | 13 | 40 | 22 | 5 | 47 | 6 | 6 | 37 | 9 | 16 | 51 | 14 | 11 | 1 | 10 | 21 | **I** |
| **94** | **L** | 48 | 51 | 71 | 70 | 15 | 90 | 68 | 14 | 78 | 1 | 10 | 67 | 40 | 67 | 65 | 52 | 45 | 22 | 45 | 34 | ***** |
| **95** | **M** | 12 | 21 | 24 | 28 | 7 | 59 | 34 | 2 | 41 | 5 | 1 | 21 | 13 | 24 | 40 | 16 | 15 | 7 | 19 | 10 | **I** |
| **96** | **L** | 4 | 14 | 41 | 29 | 8 | 52 | 37 | 5 | 47 | 1 | 8 | 38 | 14 | 34 | 45 | 18 | 15 | 6 | 20 | 24 | **I** |
| **97** | **S** | 6 | 17 | 29 | 21 | 15 | 27 | 33 | 20 | 45 | 16 | 19 | 21 | 19 | 29 | 46 | 2 | 15 | 20 | 29 | 24 | ***** |
| **98** | **R** | 47 | 63 | 73 | 44 | 55 | 76 | 42 | 51 | 38 | 41 | 39 | 32 | 40 | 35 | 1 | 44 | 41 | 52 | 66 | 43 | ***** |
| **99** | **V** | 8 | 16 | 40 | 36 | 8 | 50 | 39 | 3 | 49 | 6 | 9 | 41 | 14 | 36 | 53 | 17 | 15 | 1 | 34 | 24 | ***** |
| **100** | **I** | 15 | 16 | 38 | 36 | 9 | 51 | 36 | 1 | 46 | 2 | 9 | 36 | 10 | 28 | 44 | 26 | 18 | 5 | 33 | 19 | **L** |
| **101** | **G** | 6 | 23 | 15 | 23 | 21 | 1 | 9 | 22 | 32 | 23 | 16 | 9 | 12 | 8 | 31 | 8 | 12 | 21 | 14 | 25 | ***** |
| **102** | **G** | 18 | 60 | 55 | 58 | 58 | 1 | 55 | 54 | 65 | 53 | 54 | 41 | 33 | 58 | 52 | 32 | 56 | 61 | 41 | 63 | ***** |
| **103** | **M** | 7 | 10 | 29 | 28 | 2 | 42 | 24 | 3 | 30 | 4 | 1 | 25 | 9 | 19 | 28 | 10 | 12 | 4 | 19 | 19 | **F** |
| **104** | **S** | 8 | 11 | 23 | 21 | 11 | 16 | 26 | 22 | 31 | 18 | 13 | 17 | 12 | 19 | 43 | 1 | 10 | 19 | 29 | 31 | ***** |
| **105** | **A** | 2 | 16 | 39 | 27 | 30 | 26 | 22 | 24 | 39 | 22 | 21 | 24 | 17 | 36 | 49 | 11 | 18 | 17 | 30 | 34 | ***** |
| **106** | **G** | 9 | 32 | 42 | 49 | 55 | 1 | 31 | 59 | 56 | 58 | 44 | 29 | 30 | 49 | 59 | 15 | 36 | 46 | 51 | 58 | ***** |
| **107** | **M** | 12 | 26 | 36 | 43 | 13 | 41 | 33 | 12 | 47 | 8 | 1 | 23 | 14 | 27 | 39 | 16 | 19 | 13 | 34 | 27 | ***** |
| **108** | **V** | 13 | 14 | 40 | 36 | 8 | 48 | 37 | 4 | 40 | 10 | 8 | 30 | 15 | 27 | 55 | 17 | 10 | 2 | 30 | 13 | ***** |
| **109** | **M** | 18 | 36 | 54 | 36 | 15 | 55 | 41 | 12 | 53 | 13 | 1 | 33 | 15 | 23 | 39 | 18 | 20 | 13 | 17 | 24 | ***** |
| **110** | **P** | 47 | 83 | 77 | 74 | 84 | 84 | 81 | 71 | 85 | 81 | 73 | 76 | 1 | 70 | 90 | 52 | 47 | 53 | 87 | 86 | ***** |
| **111** | **G** | 6 | 20 | 34 | 37 | 32 | 2 | 34 | 19 | 42 | 24 | 26 | 17 | 10 | 25 | 39 | 9 | 14 | 14 | 31 | 41 | ***** |
| **112** | **V** | 10 | 16 | 58 | 52 | 27 | 63 | 48 | 8 | 68 | 17 | 17 | 54 | 19 | 44 | 72 | 18 | 18 | 2 | 53 | 28 | ***** |
| **113** | **T** | 12 | 23 | 30 | 23 | 15 | 47 | 24 | 16 | 29 | 11 | 8 | 14 | 11 | 15 | 20 | 9 | 1 | 13 | 22 | 20 | ***** |
| **114** | **G** | 3 | 32 | 34 | 40 | 38 | 2 | 34 | 28 | 39 | 34 | 23 | 18 | 15 | 35 | 42 | 8 | 14 | 18 | 34 | 34 | ***** |
| **115** | **L** | 21 | 18 | 58 | 52 | 13 | 84 | 47 | 8 | 62 | 2 | 3 | 53 | 25 | 45 | 56 | 27 | 24 | 13 | 22 | 10 | **M** |
| **116** | **I** | 19 | 49 | 71 | 70 | 36 | 78 | 71 | 2 | 78 | 19 | 19 | 70 | 36 | 70 | 77 | 31 | 41 | 7 | 69 | 49 | ***** |
| **117** | **A** | 2 | 36 | 51 | 48 | 58 | 38 | 61 | 31 | 61 | 44 | 48 | 56 | 23 | 54 | 51 | 21 | 25 | 23 | 63 | 51 | ***** |
| **118** | **D** | 32 | 57 | 1 | 15 | 58 | 72 | 33 | 55 | 59 | 53 | 50 | 30 | 37 | 29 | 60 | 26 | 37 | 49 | 61 | 53 | ***** |
| **119** | **I** | 22 | 20 | 32 | 39 | 20 | 68 | 26 | 4 | 35 | 9 | 12 | 26 | 21 | 30 | 29 | 18 | 19 | 3 | 32 | 17 | ***** |
| **120** | **S** | 20 | 39 | 31 | 31 | 23 | 62 | 37 | 30 | 51 | 37 | 36 | 35 | 30 | 37 | 58 | 3 | 12 | 25 | 41 | 27 | ***** |
| **121** | **P** | 21 | 41 | 20 | 18 | 43 | 54 | 26 | 41 | 28 | 36 | 32 | 28 | 1 | 22 | 32 | 18 | 16 | 38 | 43 | 43 | ***** |

**Table ST1. *Continuation.***

| ***Sau* NorAI** | | **Alternative aminoacid** | | | | | | | | | | | | | | | | | | | | ***Sep***  **NorA** |
| --- | --- | --- | --- | --- | --- | --- | --- | --- | --- | --- | --- | --- | --- | --- | --- | --- | --- | --- | --- | --- | --- | --- |
|  |  | **A** | **C** | **D** | **E** | **F** | **G** | **H** | **I** | **K** | **L** | **M** | **N** | **P** | **Q** | **R** | **S** | **T** | **V** | **W** | **Y** |  |
| **122** | **S** | 2 | 6 | 4 | 3 | 7 | 13 | 5 | 6 | 6 | 6 | 5 | 4 | 2 | 5 | 9 | 1 | 5 | 7 | 9 | 6 | **G** |
| **123** | **H** | 4 | 8 | 4 | 2 | 7 | 19 | 1 | 7 | 3 | 7 | 7 | 4 | 3 | 4 | 6 | 4 | 5 | 7 | 8 | 5 | **A** |
| **124** | **Q** | 14 | 24 | 4 | 4 | 23 | 51 | 9 | 19 | 9 | 20 | 11 | 8 | 10 | 1 | 9 | 10 | 11 | 20 | 19 | 15 | **D** |
| **125** | **K** | 9 | 24 | 20 | 10 | 17 | 49 | 14 | 18 | 1 | 11 | 7 | 11 | 10 | 8 | 1 | 11 | 13 | 12 | 13 | 16 | ***** |
| **126** | **A** | 2 | 29 | 45 | 38 | 39 | 17 | 43 | 24 | 51 | 27 | 25 | 27 | 9 | 28 | 43 | 11 | 15 | 24 | 49 | 44 | ***** |
| **127** | **K** | 12 | 28 | 31 | 10 | 21 | 44 | 12 | 27 | 1 | 21 | 12 | 12 | 14 | 11 | 4 | 13 | 13 | 24 | 18 | 15 | ***** |
| **128** | **N** | 6 | 24 | 18 | 19 | 19 | 15 | 10 | 13 | 22 | 15 | 12 | 1 | 15 | 16 | 15 | 8 | 10 | 11 | 18 | 12 | ***** |
| **129** | **F** | 35 | 40 | 51 | 51 | 1 | 72 | 41 | 9 | 51 | 10 | 6 | 31 | 29 | 35 | 49 | 29 | 32 | 19 | 28 | 9 | ***** |
| **130** | **G** | 13 | 49 | 48 | 51 | 56 | 1 | 47 | 55 | 58 | 56 | 45 | 22 | 29 | 51 | 60 | 11 | 31 | 50 | 48 | 54 | ***** |
| **131** | **Y** | 28 | 39 | 55 | 48 | 8 | 73 | 21 | 14 | 38 | 12 | 21 | 34 | 33 | 27 | 37 | 29 | 25 | 18 | 8 | 1 | ***** |
| **132** | **M** | 18 | 30 | 47 | 40 | 11 | 73 | 26 | 7 | 41 | 7 | 1 | 27 | 20 | 21 | 30 | 19 | 21 | 8 | 16 | 13 | ***** |
| **133** | **S** | 9 | 22 | 20 | 15 | 18 | 21 | 22 | 21 | 34 | 22 | 11 | 9 | 14 | 14 | 27 | 1 | 8 | 20 | 29 | 19 | ***** |
| **134** | **A** | 2 | 24 | 44 | 38 | 30 | 34 | 42 | 17 | 53 | 18 | 12 | 41 | 16 | 39 | 52 | 11 | 16 | 14 | 32 | 38 | ***** |
| **135** | **I** | 5 | 11 | 34 | 34 | 9 | 30 | 32 | 1 | 44 | 8 | 7 | 32 | 13 | 34 | 42 | 8 | 12 | 4 | 28 | 20 | ***** |
| **136** | **I** | 16 | 25 | 46 | 44 | 8 | 55 | 28 | 2 | 52 | 8 | 7 | 25 | 21 | 31 | 42 | 17 | 20 | 6 | 25 | 20 | ***** |
| **137** | **N** | 12 | 29 | 20 | 19 | 22 | 19 | 16 | 22 | 29 | 21 | 19 | 1 | 13 | 19 | 24 | 7 | 8 | 20 | 34 | 20 | ***** |
| **138** | **S** | 4 | 8 | 13 | 11 | 6 | 20 | 13 | 5 | 21 | 6 | 4 | 10 | 6 | 13 | 25 | 1 | 4 | 5 | 14 | 15 | ***** |
| **139** | **G** | 13 | 43 | 48 | 53 | 49 | 1 | 50 | 54 | 57 | 57 | 48 | 39 | 26 | 51 | 59 | 16 | 37 | 41 | 51 | 57 | ***** |
| **140** | **F** | 27 | 38 | 51 | 49 | 1 | 52 | 35 | 16 | 56 | 15 | 11 | 37 | 21 | 32 | 52 | 27 | 22 | 19 | 15 | 12 | ***** |
| **141** | **I** | 12 | 27 | 43 | 46 | 22 | 49 | 45 | 1 | 55 | 10 | 9 | 45 | 21 | 45 | 56 | 18 | 18 | 5 | 42 | 32 | ***** |
| **142** | **L** | 13 | 14 | 44 | 39 | 9 | 58 | 38 | 4 | 50 | 1 | 7 | 39 | 17 | 37 | 45 | 22 | 16 | 7 | 30 | 25 | ***** |
| **143** | **G** | 16 | 44 | 45 | 46 | 29 | 1 | 44 | 51 | 53 | 49 | 42 | 35 | 23 | 47 | 52 | 20 | 40 | 47 | 39 | 31 | ***** |
| **144** | **P** | 37 | 68 | 61 | 55 | 55 | 76 | 63 | 53 | 72 | 56 | 37 | 63 | 1 | 47 | 74 | 43 | 53 | 44 | 70 | 51 | ***** |
| **145** | **G** | 4 | 15 | 17 | 20 | 9 | 2 | 16 | 9 | 22 | 9 | 8 | 11 | 4 | 17 | 23 | 6 | 10 | 8 | 14 | 18 | ***** |
| **146** | **I** | 13 | 27 | 48 | 48 | 14 | 66 | 47 | 1 | 56 | 6 | 12 | 44 | 22 | 47 | 55 | 27 | 27 | 6 | 43 | 29 | **F** |
| **147** | **G** | 19 | 38 | 51 | 53 | 53 | 1 | 50 | 46 | 60 | 53 | 54 | 37 | 31 | 51 | 62 | 17 | 37 | 50 | 51 | 57 | ***** |
| **148** | **G** | 16 | 48 | 45 | 50 | 41 | 1 | 39 | 45 | 54 | 34 | 42 | 31 | 17 | 48 | 56 | 19 | 29 | 37 | 42 | 54 | ***** |
| **149** | **F** | 19 | 34 | 46 | 41 | 1 | 56 | 22 | 13 | 55 | 10 | 12 | 43 | 22 | 29 | 45 | 23 | 23 | 11 | 6 | 6 | ***** |
| **150** | **M** | 13 | 25 | 40 | 37 | 9 | 62 | 35 | 4 | 42 | 1 | 1 | 37 | 14 | 28 | 41 | 23 | 17 | 7 | 17 | 20 | **L** |
| **151** | **A** | 2 | 26 | 34 | 32 | 27 | 26 | 29 | 27 | 41 | 27 | 22 | 32 | 13 | 33 | 47 | 12 | 15 | 14 | 35 | 28 | ***** |
| **152** | **E** | 10 | 26 | 4 | 1 | 21 | 28 | 7 | 19 | 16 | 16 | 15 | 13 | 6 | 6 | 21 | 7 | 12 | 14 | 15 | 17 | ***** |
| **153** | **V** | 10 | 17 | 19 | 19 | 3 | 45 | 14 | 3 | 28 | 6 | 8 | 19 | 8 | 23 | 36 | 12 | 10 | 2 | 7 | 7 | **I** |
| **154** | **S** | 11 | 21 | 10 | 13 | 20 | 12 | 12 | 18 | 25 | 25 | 20 | 11 | 13 | 16 | 32 | 1 | 13 | 22 | 16 | 20 | ***** |
| **155** | **H** | 9 | 17 | 11 | 10 | 4 | 20 | 0 | 6 | 20 | 6 | 6 | 6 | 5 | 9 | 12 | 6 | 10 | 11 | 2 | 1 | ***** |
| **156** | **R** | 22 | 45 | 40 | 25 | 33 | 54 | 12 | 35 | 12 | 30 | 30 | 21 | 22 | 17 | 1 | 21 | 20 | 41 | 35 | 21 | ***** |

**Table ST1. *Continuation.***

| ***Sau* NorAI** | | | **Alternative aminoacid** | | | | | | | | | | | | | | | | | | | | | | | | | | | | | | | ***Sep***  **NorA** |
| --- | --- | --- | --- | --- | --- | --- | --- | --- | --- | --- | --- | --- | --- | --- | --- | --- | --- | --- | --- | --- | --- | --- | --- | --- | --- | --- | --- | --- | --- | --- | --- | --- | --- | --- |
|  |  |  | **A** | | **C** | | **D** | | **E** | | **F** | | **G** | | **H** | | **I** | | **K** | | **L** | | **M** | | **N** | **P** | **Q** | **R** | **S** | **T** | **V** | **W** | **Y** |  |
| **157** | **M** | 3 | | 16 | | 23 | | 21 | | 7 | | 40 | | 20 | | 8 | | 24 | | 6 | | 1 | | 14 | | 7 | 13 | 22 | 8 | 8 | 6 | 6 | 8 | **L** |
| **158** | **P** | 42 | | 70 | | 68 | | 66 | | 80 | | 85 | | 74 | | 56 | | 80 | | 60 | | 56 | | 75 | | 1 | 71 | 85 | 51 | 51 | 47 | 74 | 78 | ***** |
| **159** | **F** | 41 | | 46 | | 71 | | 71 | | 1 | | 71 | | 63 | | 36 | | 80 | | 25 | | 34 | | 69 | | 37 | 73 | 79 | 46 | 61 | 35 | 32 | 21 | ***** |
| **160** | **Y** | 21 | | 33 | | 46 | | 45 | | 5 | | 59 | | 18 | | 22 | | 54 | | 14 | | 19 | | 32 | | 26 | 33 | 53 | 37 | 30 | 18 | 5 | 1 | ***** |
| **161** | **F** | 13 | | 17 | | 47 | | 47 | | 1 | | 45 | | 35 | | 9 | | 47 | | 10 | | 13 | | 46 | | 26 | 37 | 55 | 16 | 20 | 10 | 16 | 12 | **V** |
| **162** | **A** | 2 | | 13 | | 44 | | 42 | | 35 | | 46 | | 38 | | 24 | | 58 | | 24 | | 21 | | 27 | | 16 | 33 | 57 | 14 | 18 | 18 | 35 | 26 | ***** |
| **163** | **G** | 9 | | 29 | | 44 | | 48 | | 31 | | 2 | | 43 | | 36 | | 54 | | 35 | | 41 | | 35 | | 22 | 47 | 54 | 13 | 23 | 28 | 36 | 47 | ***** |
| **164** | **A** | 2 | | 9 | | 25 | | 27 | | 13 | | 23 | | 28 | | 8 | | 37 | | 9 | | 14 | | 24 | | 4 | 26 | 38 | 9 | 6 | 7 | 23 | 25 | **T** |
| **165** | **L** | 18 | | 21 | | 60 | | 56 | | 16 | | 77 | | 55 | | 8 | | 65 | | 2 | | 12 | | 57 | | 18 | 44 | 60 | 30 | 26 | 11 | 34 | 35 | ***** |
| **166** | **G** | 11 | | 23 | | 42 | | 42 | | 30 | | 2 | | 43 | | 36 | | 54 | | 33 | | 26 | | 24 | | 20 | 46 | 57 | 13 | 26 | 36 | 43 | 34 | ***** |
| **167** | **I** | 10 | | 16 | | 44 | | 44 | | 8 | | 50 | | 42 | | 1 | | 53 | | 5 | | 12 | | 42 | | 13 | 42 | 53 | 17 | 18 | 3 | 31 | 26 | **V** |
| **168** | **L** | 14 | | 18 | | 56 | | 50 | | 12 | | 66 | | 49 | | 5 | | 62 | | 1 | | 9 | | 41 | | 14 | 45 | 56 | 27 | 21 | 8 | 33 | 26 | **V** |
| **169** | **A** | 2 | | 12 | | 38 | | 40 | | 29 | | 31 | | 34 | | 21 | | 55 | | 20 | | 23 | | 24 | | 13 | 39 | 50 | 10 | 18 | 16 | 30 | 27 | ***** |
| **170** | **F** | 14 | | 27 | | 57 | | 55 | | 1 | | 60 | | 44 | | 16 | | 67 | | 12 | | 15 | | 51 | | 31 | 56 | 65 | 25 | 21 | 17 | 11 | 17 | ***** |
| **171** | **I** | 18 | | 20 | | 60 | | 57 | | 17 | | 65 | | 54 | | 1 | | 60 | | 7 | | 17 | | 48 | | 17 | 59 | 60 | 38 | 29 | 6 | 31 | 32 | ***** |
| **172** | **M** | 12 | | 17 | | 48 | | 38 | | 10 | | 49 | | 41 | | 7 | | 41 | | 5 | | 1 | | 32 | | 13 | 29 | 38 | 20 | 16 | 7 | 18 | 19 | ***** |
| **173** | **S** | 6 | | 12 | | 24 | | 18 | | 15 | | 33 | | 24 | | 12 | | 33 | | 14 | | 12 | | 17 | | 13 | 22 | 41 | 1 | 7 | 12 | 25 | 24 | ***** |
| **174** | **I** | 10 | | 14 | | 41 | | 31 | | 9 | | 61 | | 31 | | 4 | | 48 | | 3 | | 8 | | 29 | | 13 | 35 | 38 | 17 | 15 | 2 | 10 | 10 | **V** |
| **175** | **V** | 10 | | 15 | | 39 | | 35 | | 7 | | 58 | | 29 | | 5 | | 31 | | 3 | | 10 | | 30 | | 10 | 27 | 25 | 15 | 11 | 2 | 15 | 15 | **L** |
| **176** | **L** | 23 | | 22 | | 40 | | 41 | | 10 | | 67 | | 45 | | 8 | | 40 | | 2 | | 10 | | 47 | | 24 | 36 | 36 | 26 | 24 | 12 | 16 | 18 | ***** |
| **177** | **I** | 33 | | 43 | | 62 | | 57 | | 23 | | 83 | | 59 | | 2 | | 52 | | 8 | | 16 | | 53 | | 19 | 45 | 60 | 39 | 36 | 9 | 39 | 33 | ***** |
| **178** | **H** | 15 | | 28 | | 16 | | 9 | | 18 | | 38 | | 1 | | 22 | | 10 | | 19 | | 25 | | 10 | | 5 | 11 | 11 | 14 | 17 | 23 | 24 | 11 | ***** |
| **179** | **D** | 11 | | 18 | | 2 | | 3 | | 16 | | 36 | | 10 | | 17 | | 17 | | 14 | | 17 | | 5 | | 6 | 11 | 21 | 8 | 12 | 15 | 18 | 16 | **N** |
| **180** | **P** | 22 | | 41 | | 23 | | 18 | | 35 | | 53 | | 29 | | 30 | | 27 | | 28 | | 25 | | 31 | | 2 | 27 | 37 | 17 | 19 | 31 | 38 | 36 | ***** |
| **181** | **K** | 10 | | 16 | | 14 | | 8 | | 15 | | 32 | | 8 | | 14 | | 2 | | 13 | | 10 | | 7 | | 6 | 8 | 3 | 10 | 10 | 13 | 13 | 13 | **Q** |
| **182** | **K** | 11 | | 17 | | 17 | | 10 | | 15 | | 28 | | 10 | | 14 | | 2 | | 13 | | 12 | | 9 | | 6 | 9 | 5 | 9 | 10 | 13 | 14 | 14 | ***** |
| **183** | **S** | 6 | | 9 | | 9 | | 4 | | 11 | | 24 | | 6 | | 10 | | 10 | | 9 | | 9 | | 7 | | 6 | 8 | 11 | 3 | 7 | 10 | 12 | 12 | **A** |
| **184** | **T** | 6 | | 11 | | 12 | | 9 | | 10 | | 20 | | 11 | | 9 | | 10 | | 10 | | 8 | | 9 | | 6 | 9 | 11 | 7 | 2 | 8 | 14 | 14 | ***** |
| **185** | **T** | 7 | | 12 | | 13 | | 10 | | 12 | | 24 | | 10 | | 10 | | 11 | | 10 | | 11 | | 9 | | 6 | 12 | 11 | 7 | 2 | 9 | 14 | 12 | ***** |
| **186** | **S** | 5 | | 9 | | 8 | | 4 | | 9 | | 22 | | 7 | | 9 | | 10 | | 9 | | 7 | | 7 | | 6 | 7 | 11 | 2 | 6 | 8 | 11 | 11 | **D** |
| **187** | **G** | 7 | | 14 | | 13 | | 12 | | 14 | | 3 | | 11 | | 13 | | 13 | | 13 | | 13 | | 9 | | 7 | 11 | 11 | 7 | 12 | 14 | 13 | 15 | ***** |
| **188** | **F** | 12 | | 18 | | 21 | | 17 | | 3 | | 31 | | 13 | | 11 | | 22 | | 10 | | 11 | | 20 | | 10 | 18 | 16 | 12 | 14 | 11 | 10 | 9 | ***** |
| **189** | **Q** | 7 | | 12 | | 7 | | 6 | | 11 | | 30 | | 5 | | 11 | | 9 | | 9 | | 7 | | 8 | | 6 | 3 | 9 | 6 | 8 | 9 | 10 | 10 | **H** |
| **190** | **K** | 9 | | 13 | | 14 | | 9 | | 11 | | 30 | | 9 | | 12 | | 3 | | 11 | | 9 | | 7 | | 6 | 8 | 6 | 5 | 8 | 11 | 9 | 9 | **Q** |

**Table ST1. *Cont.***

| ***Sau* NorAI** | | **Alternative aminoacid** | | | | | | | | | | | | | | | | | | | | | | | | | | ***Sep***  **NorA** |
| --- | --- | --- | --- | --- | --- | --- | --- | --- | --- | --- | --- | --- | --- | --- | --- | --- | --- | --- | --- | --- | --- | --- | --- | --- | --- | --- | --- | --- |
|  |  | **A** | **C** | **D** | **E** | **F** | **G** | **H** | **I** | **K** | **L** | **M** | **N** | **P** | **Q** | **R** | | **S** | | **T** | | **V** | | **W** | | **Y** | |  |
| **191** | **L** | 16 | 22 | 27 | 24 | 13 | 55 | 29 | 8 | 29 | 2 | 9 | 29 | 11 | 21 | | 27 | | 19 | | 17 | | 10 | | 19 | | 18 | **Y** |
| **192** | **E** | 13 | 29 | 8 | 2 | 19 | 41 | 13 | 21 | 17 | 14 | 17 | 15 | 8 | 9 | | 22 | | 11 | | 19 | | 15 | | 21 | | 24 | **Q** |
| **193** | **P** | 12 | 25 | 18 | 15 | 22 | 37 | 18 | 20 | 18 | 18 | 18 | 18 | 1 | 15 | | 24 | | 12 | | 15 | | 16 | | 20 | | 26 | ***** |
| **194** | **Q** | 7 | 13 | 7 | 3 | 10 | 34 | 7 | 12 | 9 | 10 | 7 | 7 | 4 | 2 | | 10 | | 6 | | 9 | | 9 | | 11 | | 10 | **E** |
| **195** | **L** | 19 | 22 | 35 | 35 | 10 | 64 | 32 | 9 | 39 | 2 | 8 | 30 | 13 | 27 | | 35 | | 25 | | 22 | | 6 | | 19 | | 20 | ***** |
| **196** | **L** | 14 | 22 | 30 | 26 | 6 | 55 | 26 | 7 | 25 | 2 | 7 | 33 | 14 | 21 | | 22 | | 16 | | 17 | | 10 | | 13 | | 19 | **F** |
| **197** | **T** | 8 | 14 | 14 | 13 | 13 | 28 | 13 | 11 | 13 | 11 | 11 | 10 | 7 | 14 | | 13 | | 8 | | 2 | | 9 | | 16 | | 12 | ***** |
| **198** | **K** | 14 | 28 | 21 | 15 | 24 | 42 | 17 | 18 | 2 | 18 | 15 | 15 | 10 | 12 | | 9 | | 13 | | 16 | | 17 | | 29 | | 19 | ***** |
| **199** | **I** | 22 | 30 | 42 | 39 | 14 | 58 | 39 | 2 | 44 | 7 | 15 | 40 | 19 | 32 | | 38 | | 23 | | 24 | | 8 | | 33 | | 21 | ***** |
| **200** | **N** | 8 | 13 | 8 | 9 | 11 | 17 | 6 | 11 | 7 | 11 | 9 | 1 | 8 | 8 | | 6 | | 6 | | 7 | | 12 | | 14 | | 10 | ***** |
| **201** | **W** | 25 | 37 | 27 | 25 | 19 | 51 | 21 | 33 | 37 | 26 | 24 | 25 | 29 | 28 | | 28 | | 25 | | 29 | | 30 | | 1 | | 18 | ***** |
| **202** | **K** | 15 | 28 | 24 | 15 | 19 | 36 | 16 | 20 | 2 | 19 | 15 | 15 | 6 | 12 | | 5 | | 13 | | 13 | | 20 | | 25 | | 18 | ***** |
| **203** | **V** | 10 | 17 | 22 | 18 | 15 | 45 | 22 | 6 | 27 | 8 | 9 | 22 | 6 | 17 | | 28 | | 14 | | 12 | | 2 | | 20 | | 15 | ***** |
| **204** | **F** | 29 | 55 | 64 | 54 | 2 | 71 | 41 | 18 | 65 | 16 | 27 | 51 | 32 | 62 | | 61 | | 41 | | 40 | | 22 | | 26 | | 11 | ***** |
| **205** | **I** | 21 | 25 | 49 | 52 | 12 | 58 | 51 | 2 | 46 | 8 | 13 | 39 | 21 | 43 | | 34 | | 27 | | 22 | | 7 | | 33 | | 28 | ***** |
| **206** | **T** | 8 | 14 | 23 | 19 | 13 | 31 | 19 | 9 | 21 | 10 | 11 | 15 | 7 | 20 | | 15 | | 7 | | 2 | | 8 | | 18 | | 16 | ***** |
| **207** | **P** | 20 | 37 | 49 | 43 | 38 | 66 | 39 | 35 | 54 | 26 | 26 | 50 | 1 | 43 | | 55 | | 29 | | 29 | | 31 | | 47 | | 37 | ***** |
| **208** | **V** | 3 | 10 | 26 | 23 | 7 | 53 | 22 | 3 | 33 | 5 | 6 | 24 | 8 | 22 | | 36 | | 11 | | 10 | | 3 | | 16 | | 10 | ***** |
| **209** | **I** | 21 | 26 | 60 | 55 | 16 | 62 | 62 | 2 | 63 | 8 | 17 | 58 | 25 | 60 | | 60 | | 32 | | 27 | | 8 | | 38 | | 31 | ***** |
| **210** | **L** | 16 | 16 | 57 | 52 | 14 | 62 | 48 | 6 | 53 | 2 | 9 | 40 | 18 | 37 | | 46 | | 25 | | 21 | | 10 | | 27 | | 23 | ***** |
| **211** | **T** | 12 | 19 | 34 | 34 | 18 | 41 | 29 | 13 | 39 | 17 | 13 | 19 | 13 | 24 | | 32 | | 11 | | 2 | | 11 | | 24 | | 21 | ***** |
| **212** | **L** | 21 | 25 | 61 | 55 | 7 | 57 | 54 | 9 | 67 | 2 | 8 | 43 | 25 | 48 | | 61 | | 27 | | 22 | | 14 | | 36 | | 31 | ***** |
| **213** | **V** | 16 | 17 | 62 | 42 | 17 | 62 | 53 | 6 | 69 | 10 | 12 | 55 | 23 | 41 | | 73 | | 29 | | 21 | | 2 | | 34 | | 32 | ***** |
| **214** | **L** | 18 | 18 | 53 | 46 | 12 | 65 | 32 | 8 | 51 | 2 | 7 | 32 | 18 | 31 | | 43 | | 21 | | 20 | | 10 | | 21 | | 17 | ***** |
| **215** | **S** | 2 | 11 | 16 | 13 | 10 | 29 | 10 | 13 | 24 | 13 | 8 | 8 | 9 | 10 | | 26 | | 3 | | 7 | | 13 | | 13 | | 13 | **A** |
| **216** | **F** | 29 | 40 | 66 | 62 | 2 | 71 | 44 | 20 | 74 | 17 | 22 | 48 | 42 | 66 | | 73 | | 36 | | 32 | | 27 | | 22 | | 16 | ***** |
| **217** | **G** | 16 | 32 | 49 | 53 | 41 | 2 | 51 | 42 | 67 | 42 | 36 | 41 | 28 | 58 | | 64 | | 20 | | 34 | | 35 | | 34 | | 48 | ***** |
| **218** | **L** | 20 | 21 | 45 | 39 | 10 | 60 | 25 | 8 | 58 | 2 | 6 | 29 | 22 | 27 | | 43 | | 25 | | 21 | | 13 | | 18 | | 13 | ***** |
| **219** | **S** | 8 | 22 | 28 | 22 | 14 | 32 | 24 | 19 | 38 | 22 | 11 | 17 | 17 | 18 | | 42 | | 2 | | 11 | | 19 | | 30 | | 17 | ***** |
| **220** | **A** | 2 | 13 | 48 | 39 | 29 | 27 | 43 | 19 | 50 | 23 | 19 | 26 | 13 | 20 | | 56 | | 13 | | 17 | | 16 | | 37 | | 32 | ***** |
| **221** | **F** | 33 | 38 | 64 | 65 | 2 | 77 | 41 | 19 | 73 | 19 | 17 | 58 | 37 | 52 | | 58 | | 39 | | 35 | | 23 | | 21 | | 9 | ***** |
| **222** | **E** | 18 | 37 | 10 | 2 | 15 | 49 | 17 | 19 | 34 | 20 | 15 | 17 | 9 | 8 | | 35 | | 13 | | 16 | | 17 | | 20 | | 16 | ***** |
| **223** | **T** | 13 | 30 | 46 | 35 | 25 | 48 | 43 | 21 | 49 | 23 | 22 | 22 | 11 | 29 | | 50 | | 10 | | 2 | | 16 | | 40 | | 29 | ***** |
| **224** | **L** | 16 | 16 | 49 | 43 | 7 | 55 | 36 | 6 | 53 | 2 | 7 | 29 | 16 | 26 | | 46 | | 19 | | 12 | | 7 | | 17 | | 9 | ***** |
| **225** | **Y** | 9 | 16 | 28 | 19 | 1 | 43 | 9 | 6 | 28 | 4 | 7 | 20 | 8 | 17 | | 27 | | 11 | | 11 | | 7 | | 4 | | 1 | **F** |

**Table ST1. *Cont.***

| ***Sau* NorAI** | | **Alternative aminoacid** | | | | | | | | | | | | | | | | | | | | | | | | | | ***Sep***  **NorA** |
| --- | --- | --- | --- | --- | --- | --- | --- | --- | --- | --- | --- | --- | --- | --- | --- | --- | --- | --- | --- | --- | --- | --- | --- | --- | --- | --- | --- | --- |
|  |  | **A** | **C** | **D** | **E** | **F** | **G** | **H** | **I** | **K** | **L** | **M** | **N** | **P** | **Q** | **R** | | **S** | | **T** | | **V** | | **W** | | **Y** | |  |
| **226** | **S** | 12 | 39 | 33 | 30 | 36 | 37 | 44 | 31 | 46 | 36 | 25 | 21 | 7 | 30 | | 61 | | 2 | | 14 | | 28 | | 45 | | 43 | ***** |
| **227** | **L** | 31 | 44 | 52 | 49 | 17 | 78 | 59 | 13 | 70 | 2 | 16 | 60 | 16 | 41 | | 56 | | 45 | | 36 | | 17 | | 44 | | 25 | ***** |
| **228** | **Y** | 50 | 67 | 74 | 67 | 11 | 88 | 25 | 39 | 73 | 35 | 36 | 67 | 53 | 54 | | 59 | | 53 | | 60 | | 38 | | 19 | | 1 | ***** |
| **229** | **T** | 10 | 21 | 38 | 28 | 14 | 42 | 38 | 13 | 37 | 9 | 12 | 23 | 15 | 28 | | 39 | | 11 | | 2 | | 9 | | 25 | | 22 | ***** |
| **230** | **A** | 2 | 10 | 11 | 9 | 14 | 24 | 10 | 7 | 15 | 9 | 8 | 11 | 5 | 8 | | 16 | | 2 | | 6 | | 7 | | 15 | | 12 | ***** |
| **231** | **D** | 13 | 19 | 2 | 3 | 16 | 40 | 8 | 16 | 24 | 18 | 18 | 9 | 9 | 8 | | 31 | | 10 | | 13 | | 19 | | 20 | | 5 | ***** |
| **232** | **K** | 14 | 24 | 23 | 12 | 18 | 38 | 13 | 17 | 2 | 16 | 13 | 10 | 12 | 11 | | 6 | | 12 | | 13 | | 14 | | 25 | | 15 | ***** |
| **233** | **V** | 5 | 16 | 23 | 17 | 6 | 32 | 12 | 6 | 23 | 6 | 7 | 23 | 9 | 20 | | 20 | | 16 | | 13 | | 2 | | 18 | | 7 | ***** |
| **234** | **N** | 10 | 16 | 9 | 9 | 15 | 6 | 5 | 15 | 10 | 12 | 12 | 2 | 9 | 8 | | 10 | | 7 | | 10 | | 17 | | 16 | | 13 | ***** |
| **235** | **Y** | 27 | 53 | 57 | 41 | 9 | 60 | 21 | 28 | 53 | 21 | 23 | 41 | 38 | 33 | | 46 | | 35 | | 38 | | 26 | | 12 | | 2 | ***** |
| **236** | **S** | 15 | 26 | 13 | 13 | 31 | 26 | 24 | 31 | 27 | 29 | 24 | 13 | 14 | 19 | | 38 | | 3 | | 6 | | 26 | | 30 | | 33 | **T** |
| **237** | **P** | 27 | 62 | 45 | 34 | 59 | 69 | 44 | 48 | 56 | 42 | 39 | 52 | 1 | 45 | | 65 | | 36 | | 36 | | 45 | | 60 | | 61 | ***** |
| **238** | **K** | 6 | 11 | 12 | 7 | 9 | 24 | 7 | 9 | 2 | 8 | 6 | 6 | 6 | 5 | | 3 | | 6 | | 6 | | 8 | | 9 | | 8 | ***** |
| **239** | **D** | 16 | 32 | 2 | 6 | 23 | 47 | 13 | 23 | 36 | 24 | 18 | 14 | 13 | 10 | | 44 | | 13 | | 17 | | 22 | | 26 | | 29 | ***** |
| **240** | **I** | 27 | 45 | 66 | 63 | 24 | 85 | 52 | 3 | 58 | 16 | 22 | 45 | 37 | 52 | | 70 | | 35 | | 31 | | 10 | | 49 | | 28 | ***** |
| **241** | **S** | 9 | 23 | 26 | 26 | 21 | 16 | 23 | 29 | 36 | 23 | 21 | 19 | 12 | 25 | | 47 | | 2 | | 12 | | 30 | | 17 | | 20 | ***** |
| **242** | **I** | 16 | 31 | 54 | 51 | 10 | 56 | 49 | 2 | 47 | 7 | 11 | 47 | 25 | 49 | | 48 | | 33 | | 24 | | 7 | | 17 | | 17 | ***** |
| **243** | **A** | 2 | 14 | 30 | 33 | 15 | 39 | 28 | 9 | 47 | 10 | 11 | 38 | 10 | 24 | | 43 | | 12 | | 15 | | 8 | | 20 | | 24 | ***** |
| **244** | **I** | 21 | 27 | 56 | 44 | 8 | 74 | 49 | 2 | 50 | 6 | 7 | 49 | 18 | 50 | | 63 | | 29 | | 23 | | 7 | | 23 | | 14 | ***** |
| **245** | **T** | 10 | 24 | 36 | 29 | 15 | 43 | 35 | 15 | 41 | 14 | 10 | 24 | 14 | 27 | | 37 | | 10 | | 2 | | 6 | | 31 | | 23 | **I** |
| **246** | **G** | 7 | 17 | 31 | 29 | 13 | 3 | 29 | 14 | 38 | 15 | 18 | 20 | 8 | 27 | | 37 | | 11 | | 17 | | 13 | | 20 | | 21 | ***** |
| **247** | **G** | 10 | 19 | 29 | 35 | 18 | 3 | 25 | 22 | 36 | 23 | 17 | 10 | 10 | 23 | | 32 | | 11 | | 19 | | 19 | | 17 | | 19 | ***** |
| **248** | **G** | 17 | 52 | 54 | 51 | 42 | 2 | 48 | 54 | 63 | 46 | 35 | 31 | 28 | 46 | | 65 | | 19 | | 32 | | 43 | | 53 | | 48 | ***** |
| **249** | **I** | 15 | 22 | 50 | 50 | 15 | 58 | 48 | 2 | 61 | 8 | 14 | 45 | 19 | 51 | | 59 | | 26 | | 24 | | 3 | | 30 | | 34 | **V** |
| **250** | **F** | 4 | 13 | 31 | 26 | 1 | 32 | 20 | 8 | 35 | 8 | 10 | 24 | 10 | 25 | | 39 | | 14 | | 14 | | 8 | | 12 | | 9 | ***** |
| **251** | **G** | 13 | 38 | 37 | 46 | 34 | 2 | 33 | 35 | 53 | 33 | 24 | 23 | 28 | 40 | | 46 | | 15 | | 27 | | 32 | | 30 | | 38 | ***** |
| **252** | **A** | 2 | 24 | 54 | 48 | 25 | 41 | 52 | 18 | 63 | 21 | 22 | 35 | 14 | 42 | | 51 | | 16 | | 22 | | 15 | | 43 | | 42 | ***** |
| **253** | **L** | 20 | 24 | 47 | 44 | 11 | 65 | 43 | 6 | 56 | 2 | 8 | 40 | 16 | 33 | | 42 | | 30 | | 20 | | 5 | | 35 | | 30 | ***** |
| **254** | **F** | 16 | 22 | 49 | 48 | 2 | 47 | 38 | 11 | 60 | 11 | 14 | 37 | 20 | 42 | | 58 | | 23 | | 23 | | 11 | | 24 | | 13 | ***** |
| **255** | **Q** | 23 | 45 | 35 | 21 | 46 | 54 | 38 | 39 | 46 | 32 | 18 | 26 | 21 | 2 | | 36 | | 19 | | 23 | | 37 | | 38 | | 34 | ***** |
| **256** | **I** | 8 | 16 | 28 | 26 | 9 | 28 | 25 | 2 | 32 | 5 | 6 | 15 | 7 | 19 | | 20 | | 10 | | 10 | | 3 | | 18 | | 12 | **V** |
| **257** | **Y** | 12 | 15 | 28 | 21 | 2 | 34 | 11 | 8 | 20 | 7 | 8 | 16 | 6 | 12 | | 16 | | 12 | | 10 | | 9 | | 4 | | 1 | **F** |
| **258** | **F** | 19 | 33 | 57 | 51 | 2 | 61 | 35 | 12 | 62 | 11 | 19 | 50 | 20 | 43 | | 47 | | 32 | | 26 | | 16 | | 17 | | 13 | ***** |
| **259** | **F** | 18 | 24 | 50 | 47 | 2 | 43 | 33 | 11 | 61 | 14 | 17 | 35 | 22 | 48 | | 47 | | 20 | | 19 | | 14 | | 15 | | 10 | ***** |

**Table ST2. *Continuation.***

| ***Sau* NorAI** | | **Alternative aminoacid** | | | | | | | | | | | | | | | | | | | | ***Sep***  **NorA** |
| --- | --- | --- | --- | --- | --- | --- | --- | --- | --- | --- | --- | --- | --- | --- | --- | --- | --- | --- | --- | --- | --- | --- |
|  |  | **A** | **C** | **D** | **E** | **F** | **G** | **H** | **I** | **K** | **L** | **M** | **N** | **P** | **Q** | **R** | **S** | **T** | **V** | **W** | **Y** |  |
| **259** | **F** | 18 | 24 | 50 | 47 | 2 | 43 | 33 | 11 | 61 | 14 | 17 | 35 | 22 | 48 | 47 | 20 | 19 | 14 | 15 | 10 | ***** |
| **260** | **D** | 14 | 27 | 1 | 7 | 23 | 19 | 16 | 23 | 28 | 23 | 21 | 12 | 6 | 14 | 25 | 8 | 17 | 21 | 28 | 26 | ***** |
| **261** | **K** | 22 | 40 | 43 | 22 | 26 | 56 | 19 | 23 | 2 | 24 | 17 | 21 | 11 | 18 | 5 | 21 | 20 | 24 | 13 | 19 | ***** |
| **262** | **F** | 22 | 45 | 64 | 62 | 2 | 67 | 48 | 13 | 55 | 9 | 19 | 46 | 38 | 42 | 54 | 31 | 35 | 18 | 14 | 13 | ***** |
| **263** | **M** | 10 | 19 | 22 | 18 | 11 | 39 | 21 | 8 | 23 | 6 | 2 | 20 | 11 | 15 | 20 | 8 | 12 | 6 | 25 | 16 | ***** |
| **264** | **K** | 11 | 17 | 11 | 10 | 14 | 25 | 12 | 16 | 2 | 15 | 10 | 7 | 7 | 10 | 5 | 11 | 11 | 19 | 18 | 15 | ***** |
| **265** | **Y** | 12 | 19 | 20 | 20 | 7 | 35 | 6 | 13 | 9 | 11 | 12 | 14 | 12 | 13 | 6 | 13 | 13 | 13 | 5 | 1 | ***** |
| **266** | **F** | 22 | 27 | 36 | 29 | 2 | 38 | 19 | 13 | 27 | 12 | 7 | 23 | 18 | 32 | 27 | 20 | 22 | 17 | 12 | 8 | **M** |
| **267** | **S** | 9 | 17 | 11 | 12 | 18 | 13 | 12 | 16 | 14 | 15 | 12 | 9 | 8 | 12 | 17 | 2 | 6 | 16 | 15 | 20 | ***** |
| **268** | **E** | 19 | 36 | 12 | 2 | 26 | 49 | 16 | 27 | 22 | 25 | 22 | 20 | 8 | 14 | 23 | 15 | 23 | 24 | 34 | 26 | ***** |
| **269** | **L** | 12 | 16 | 26 | 18 | 10 | 43 | 17 | 6 | 16 | 2 | 5 | 19 | 7 | 14 | 13 | 13 | 11 | 7 | 12 | 10 | ***** |
| **270** | **T** | 12 | 20 | 31 | 30 | 22 | 39 | 22 | 16 | 18 | 16 | 16 | 15 | 8 | 20 | 14 | 13 | 2 | 13 | 17 | 23 | **N** |
| **271** | **F** | 23 | 33 | 59 | 57 | 2 | 64 | 46 | 11 | 59 | 10 | 13 | 55 | 24 | 38 | 50 | 25 | 19 | 11 | 17 | 12 | ***** |
| **272** | **I** | 32 | 40 | 74 | 72 | 24 | 76 | 71 | 2 | 78 | 12 | 15 | 60 | 42 | 56 | 66 | 42 | 34 | 11 | 69 | 46 | ***** |
| **273** | **A** | 2 | 9 | 28 | 22 | 14 | 24 | 22 | 4 | 26 | 10 | 11 | 19 | 7 | 15 | 19 | 8 | 9 | 7 | 16 | 14 | ***** |
| **274** | **W** | 16 | 22 | 52 | 40 | 10 | 40 | 35 | 15 | 48 | 13 | 18 | 41 | 18 | 35 | 45 | 24 | 21 | 18 | 1 | 12 | ***** |
| **275** | **S** | 8 | 11 | 30 | 25 | 20 | 18 | 33 | 22 | 45 | 19 | 21 | 18 | 16 | 30 | 54 | 2 | 12 | 19 | 37 | 32 | ***** |
| **276** | **L** | 27 | 27 | 69 | 63 | 16 | 73 | 50 | 13 | 72 | 2 | 11 | 49 | 33 | 47 | 67 | 36 | 34 | 19 | 50 | 32 | ***** |
| **277** | **L** | 11 | 13 | 39 | 35 | 10 | 53 | 35 | 2 | 45 | 3 | 8 | 36 | 11 | 33 | 43 | 14 | 14 | 7 | 16 | 21 | ***** |
| **278** | **Y** | 7 | 7 | 33 | 24 | 2 | 27 | 8 | 5 | 32 | 4 | 6 | 17 | 16 | 18 | 26 | 12 | 9 | 5 | 6 | 1 | ***** |
| **279** | **S** | 7 | 14 | 23 | 24 | 17 | 33 | 27 | 18 | 36 | 18 | 9 | 19 | 17 | 20 | 42 | 2 | 11 | 20 | 33 | 21 | ***** |
| **280** | **V** | 3 | 10 | 31 | 26 | 12 | 30 | 30 | 4 | 47 | 8 | 8 | 28 | 11 | 27 | 50 | 14 | 10 | 2 | 29 | 25 | **A** |
| **281** | **V** | 9 | 14 | 37 | 31 | 14 | 45 | 36 | 2 | 48 | 6 | 10 | 37 | 9 | 36 | 45 | 17 | 14 | 4 | 27 | 20 | **I** |
| **282** | **V** | 7 | 11 | 38 | 26 | 10 | 26 | 41 | 6 | 50 | 7 | 7 | 38 | 10 | 26 | 57 | 11 | 13 | 2 | 28 | 20 | ***** |
| **283** | **L** | 23 | 33 | 65 | 59 | 13 | 69 | 60 | 10 | 71 | 2 | 9 | 48 | 31 | 42 | 66 | 34 | 25 | 16 | 32 | 22 | ***** |
| **284** | **I** | 9 | 16 | 38 | 38 | 7 | 19 | 36 | 4 | 48 | 5 | 10 | 36 | 10 | 38 | 44 | 16 | 14 | 2 | 26 | 15 | **V** |
| **285** | **L** | 18 | 21 | 60 | 57 | 15 | 64 | 54 | 9 | 65 | 2 | 8 | 55 | 17 | 50 | 60 | 31 | 25 | 13 | 22 | 28 | **M** |
| **286** | **L** | 32 | 28 | 68 | 64 | 18 | 76 | 56 | 12 | 74 | 2 | 9 | 57 | 27 | 40 | 66 | 33 | 24 | 16 | 36 | 26 | ***** |
| **287** | **V** | 6 | 14 | 27 | 29 | 12 | 35 | 28 | 3 | 40 | 6 | 8 | 23 | 5 | 28 | 44 | 10 | 9 | 2 | 17 | 12 | ***** |
| **288** | **F** | 18 | 28 | 32 | 32 | 3 | 51 | 23 | 4 | 49 | 6 | 17 | 34 | 13 | 22 | 36 | 22 | 19 | 13 | 11 | 8 | **L** |
| **289** | **A** | 2 | 14 | 23 | 24 | 19 | 31 | 21 | 17 | 30 | 18 | 14 | 25 | 8 | 23 | 38 | 4 | 12 | 11 | 27 | 26 | ***** |
| **290** | **N** | 7 | 13 | 3 | 8 | 9 | 17 | 2 | 10 | 8 | 10 | 8 | 2 | 4 | 6 | 9 | 5 | 5 | 9 | 11 | 10 | ***** |
| **291** | **G** | 9 | 22 | 13 | 18 | 20 | 2 | 12 | 22 | 21 | 19 | 20 | 6 | 8 | 12 | 20 | 3 | 10 | 20 | 14 | 18 | ***** |
| **292** | **Y** | 19 | 30 | 35 | 30 | 5 | 53 | 12 | 12 | 29 | 12 | 19 | 23 | 14 | 20 | 34 | 22 | 18 | 15 | 7 | 1 | ***** |
| **293** | **W** | 35 | 68 | 76 | 57 | 34 | 66 | 65 | 40 | 78 | 32 | 44 | 73 | 30 | 54 | 77 | 48 | 34 | 43 | 1 | 27 | ***** |

**Table ST1. *Cont.***

| ***Sau* NorAI** | | **Alternative aminoacid** | | | | | | | | | | | | | | | | | | | | ***Sep***  **NorA** |
| --- | --- | --- | --- | --- | --- | --- | --- | --- | --- | --- | --- | --- | --- | --- | --- | --- | --- | --- | --- | --- | --- | --- |
|  |  | **A** | **C** | **D** | **E** | **F** | **G** | **H** | **I** | **K** | **L** | **M** | **N** | **P** | **Q** | **R** | **S** | **T** | **V** | **W** | **Y** |  |
| **294** | **S** | 5 | 10 | 13 | 9 | 10 | 26 | 9 | 10 | 21 | 7 | 6 | 10 | 5 | 9 | 23 | 3 | 3 | 8 | 8 | 13 | **T** |
| **295** | **I** | 20 | 25 | 47 | 56 | 11 | 66 | 52 | 2 | 67 | 6 | 11 | 55 | 22 | 45 | 57 | 32 | 25 | 7 | 35 | 23 | ***** |
| **296** | **M** | 14 | 24 | 45 | 45 | 11 | 52 | 32 | 8 | 51 | 6 | 2 | 46 | 15 | 36 | 52 | 23 | 20 | 9 | 19 | 15 | ***** |
| **297** | **L** | 10 | 16 | 44 | 42 | 6 | 53 | 40 | 6 | 53 | 4 | 9 | 41 | 10 | 31 | 48 | 21 | 18 | 2 | 18 | 24 | **I** |
| **298** | **I** | 12 | 17 | 52 | 54 | 16 | 54 | 52 | 2 | 62 | 9 | 14 | 49 | 15 | 54 | 60 | 18 | 18 | 6 | 37 | 23 | ***** |
| **299** | **S** | 7 | 14 | 26 | 22 | 16 | 37 | 23 | 13 | 36 | 12 | 9 | 15 | 14 | 17 | 33 | 2 | 8 | 13 | 22 | 21 | ***** |
| **300** | **F** | 27 | 27 | 68 | 67 | 2 | 67 | 55 | 20 | 77 | 21 | 24 | 67 | 33 | 62 | 74 | 34 | 30 | 21 | 28 | 21 | ***** |
| **301** | **V** | 4 | 12 | 42 | 38 | 9 | 59 | 35 | 3 | 54 | 6 | 11 | 36 | 10 | 41 | 57 | 22 | 18 | 4 | 22 | 28 | ***** |
| **302** | **V** | 15 | 22 | 52 | 37 | 9 | 58 | 46 | 5 | 63 | 9 | 9 | 37 | 19 | 31 | 65 | 25 | 20 | 2 | 25 | 16 | ***** |
| **303** | **F** | 18 | 24 | 56 | 42 | 1 | 30 | 45 | 22 | 65 | 21 | 17 | 38 | 33 | 52 | 66 | 18 | 20 | 21 | 15 | 14 | ***** |
| **304** | **I** | 21 | 24 | 63 | 46 | 16 | 65 | 58 | 2 | 70 | 8 | 14 | 47 | 33 | 52 | 68 | 26 | 21 | 9 | 40 | 34 | ***** |
| **305** | **G** | 18 | 42 | 62 | 54 | 33 | 2 | 58 | 52 | 67 | 46 | 48 | 50 | 39 | 59 | 65 | 26 | 42 | 55 | 58 | 65 | ***** |
| **306** | **F** | 24 | 36 | 42 | 34 | 2 | 58 | 30 | 15 | 59 | 19 | 15 | 25 | 33 | 36 | 60 | 23 | 23 | 19 | 17 | 11 | ***** |
| **307** | **D** | 9 | 22 | 1 | 9 | 19 | 18 | 22 | 16 | 44 | 20 | 13 | 7 | 10 | 14 | 51 | 6 | 12 | 18 | 17 | 28 | ***** |
| **308** | **M** | 16 | 23 | 48 | 45 | 13 | 49 | 39 | 9 | 48 | 4 | 2 | 37 | 17 | 32 | 43 | 19 | 21 | 12 | 23 | 29 | ***** |
| **309** | **I** | 19 | 22 | 46 | 55 | 14 | 64 | 41 | 2 | 65 | 8 | 12 | 38 | 27 | 42 | 62 | 27 | 19 | 7 | 42 | 29 | ***** |
| **310** | **R** | 18 | 29 | 37 | 20 | 18 | 44 | 14 | 21 | 14 | 16 | 11 | 12 | 16 | 12 | 2 | 17 | 15 | 25 | 21 | 17 | ***** |
| **311** | **P** | 64 | 89 | 89 | 86 | 90 | 94 | 90 | 82 | 93 | 85 | 87 | 84 | 2 | 84 | 95 | 69 | 68 | 77 | 94 | 92 | ***** |
| **312** | **A** | 3 | 18 | 63 | 55 | 40 | 50 | 58 | 32 | 68 | 31 | 32 | 37 | 16 | 51 | 70 | 16 | 20 | 25 | 64 | 57 | ***** |
| **313** | **I** | 17 | 23 | 51 | 47 | 14 | 64 | 44 | 2 | 59 | 3 | 10 | 30 | 17 | 26 | 45 | 20 | 18 | 7 | 44 | 23 | **L** |
| **314** | **T** | 23 | 41 | 40 | 44 | 40 | 65 | 51 | 29 | 54 | 30 | 20 | 18 | 13 | 21 | 48 | 14 | 2 | 25 | 50 | 39 | ***** |
| **315** | **N** | 10 | 36 | 18 | 28 | 39 | 33 | 16 | 26 | 35 | 26 | 26 | 2 | 22 | 25 | 37 | 6 | 7 | 23 | 45 | 27 | ***** |
| **316** | **Y** | 22 | 29 | 60 | 41 | 9 | 60 | 14 | 17 | 51 | 11 | 14 | 46 | 31 | 26 | 39 | 31 | 26 | 17 | 8 | 1 | ***** |
| **317** | **F** | 10 | 22 | 42 | 35 | 1 | 55 | 29 | 6 | 52 | 5 | 6 | 30 | 20 | 25 | 50 | 16 | 16 | 7 | 20 | 5 | ***** |
| **318** | **S** | 20 | 38 | 37 | 36 | 55 | 59 | 46 | 46 | 51 | 48 | 32 | 33 | 35 | 39 | 55 | 3 | 20 | 39 | 63 | 60 | ***** |
| **319** | **N** | 12 | 21 | 10 | 10 | 23 | 25 | 9 | 18 | 10 | 16 | 11 | 2 | 15 | 9 | 9 | 9 | 11 | 18 | 26 | 16 | ***** |
| **320** | **I** | 21 | 28 | 42 | 32 | 20 | 58 | 31 | 3 | 46 | 9 | 14 | 37 | 29 | 38 | 38 | 29 | 23 | 8 | 49 | 24 | ***** |
| **321** | **A** | 3 | 35 | 35 | 40 | 47 | 38 | 40 | 43 | 53 | 46 | 51 | 46 | 18 | 41 | 54 | 16 | 30 | 29 | 62 | 42 | ***** |
| **322** | **G** | 7 | 19 | 10 | 11 | 20 | 1 | 9 | 17 | 9 | 17 | 14 | 9 | 4 | 12 | 12 | 7 | 11 | 15 | 11 | 18 | ***** |
| **323** | **E** | 9 | 14 | 3 | 4 | 15 | 26 | 8 | 15 | 12 | 14 | 14 | 5 | 6 | 7 | 15 | 9 | 9 | 12 | 14 | 14 | **K** |
| **324** | **R** | 19 | 36 | 36 | 20 | 29 | 43 | 14 | 28 | 13 | 24 | 18 | 13 | 22 | 13 | 2 | 23 | 20 | 32 | 30 | 22 | ***** |
| **325** | **Q** | 32 | 55 | 43 | 24 | 56 | 66 | 27 | 41 | 28 | 47 | 24 | 42 | 26 | 2 | 17 | 28 | 31 | 40 | 54 | 33 | ***** |
| **326** | **G** | 44 | 84 | 75 | 75 | 86 | 1 | 79 | 88 | 84 | 85 | 78 | 59 | 56 | 81 | 84 | 45 | 63 | 82 | 81 | 85 | ***** |
| **327** | **F** | 13 | 28 | 42 | 26 | 2 | 50 | 23 | 11 | 35 | 10 | 11 | 38 | 23 | 26 | 32 | 14 | 13 | 14 | 12 | 9 | ***** |

**Table ST1. *Cont.***

| ***Sau* NorAI** | | | **Alternative aminoacid** | | | | | | | | | | | | | | | | | | | | ***Sep***  **NorA** |
| --- | --- | --- | --- | --- | --- | --- | --- | --- | --- | --- | --- | --- | --- | --- | --- | --- | --- | --- | --- | --- | --- | --- | --- |
|  |  |  | **A** | **C** | **D** | **E** | **F** | **G** | **H** | **I** | **K** | **L** | **M** | **N** | **P** | **Q** | **R** | **S** | **T** | **V** | **W** | **Y** |  |
| **328** | **A** | | 2 | 43 | 58 | 52 | 43 | 48 | 52 | 24 | 71 | 30 | 29 | 52 | 29 | 49 | 66 | 28 | 30 | 18 | 57 | 29 | ***** |
| **329** | **G** | | 8 | 31 | 34 | 36 | 15 | 3 | 30 | 23 | 40 | 16 | 9 | 11 | 18 | 17 | 40 | 7 | 13 | 20 | 28 | 21 | ***** |
| **330** | **G** | | 24 | 69 | 71 | 67 | 71 | 2 | 70 | 79 | 78 | 75 | 73 | 58 | 50 | 73 | 75 | 22 | 50 | 74 | 70 | 74 | ***** |
| **331** | **L** | | 24 | 30 | 65 | 61 | 17 | 71 | 57 | 11 | 62 | 3 | 11 | 61 | 34 | 57 | 63 | 34 | 25 | 12 | 31 | 28 | ***** |
| **332** | **N** | | 28 | 43 | 39 | 40 | 24 | 61 | 24 | 31 | 45 | 27 | 22 | 2 | 37 | 27 | 46 | 19 | 24 | 32 | 47 | 20 | ***** |
| **333** | **S** | | 13 | 34 | 30 | 25 | 26 | 39 | 30 | 32 | 46 | 30 | 17 | 10 | 24 | 19 | 43 | 3 | 13 | 32 | 48 | 30 | ***** |
| **334** | **T** | | 13 | 28 | 50 | 46 | 21 | 51 | 48 | 24 | 52 | 24 | 17 | 35 | 24 | 43 | 54 | 10 | 2 | 28 | 40 | 42 | ***** |
| **335** | **F** | | 17 | 21 | 56 | 52 | 2 | 57 | 42 | 14 | 63 | 14 | 16 | 51 | 28 | 51 | 64 | 22 | 25 | 20 | 24 | 13 | ***** |
| **336** | **T** | | 10 | 20 | 21 | 19 | 12 | 32 | 18 | 12 | 23 | 10 | 7 | 10 | 11 | 10 | 13 | 9 | 2 | 10 | 14 | 18 | ***** |
| **337** | **S** | | 15 | 32 | 20 | 24 | 19 | 50 | 25 | 38 | 48 | 37 | 21 | 13 | 24 | 21 | 45 | 3 | 17 | 35 | 36 | 24 | ***** |
| **338** | **M** | | 13 | 22 | 41 | 38 | 9 | 43 | 38 | 7 | 45 | 4 | 2 | 36 | 17 | 30 | 43 | 21 | 17 | 7 | 22 | 28 | ***** |
| **339** | **G** | | 18 | 43 | 58 | 61 | 54 | 1 | 58 | 50 | 66 | 53 | 42 | 47 | 36 | 60 | 68 | 27 | 37 | 48 | 56 | 62 | ***** |
| **340** | **N** | | 19 | 39 | 23 | 22 | 30 | 32 | 17 | 22 | 29 | 29 | 16 | 2 | 24 | 16 | 20 | 12 | 18 | 25 | 38 | 25 | ***** |
| **341** | **F** | | 16 | 36 | 50 | 49 | 2 | 45 | 41 | 10 | 61 | 19 | 13 | 49 | 28 | 49 | 59 | 21 | 20 | 15 | 30 | 18 | ***** |
| **342** | **I** | | 13 | 16 | 44 | 43 | 10 | 48 | 42 | 2 | 52 | 7 | 14 | 42 | 15 | 43 | 52 | 21 | 19 | 4 | 38 | 30 | ***** |
| **343** | **G** | | 21 | 57 | 62 | 55 | 55 | 1 | 63 | 65 | 69 | 65 | 55 | 43 | 36 | 64 | 71 | 25 | 44 | 60 | 56 | 66 | ***** |
| **344** | **P** | | 42 | 73 | 72 | 70 | 73 | 76 | 75 | 70 | 81 | 76 | 62 | 67 | 2 | 72 | 84 | 45 | 55 | 58 | 82 | 81 | ***** |
| **345** | | **L** | 15 | 33 | 56 | 52 | 14 | 68 | 51 | 8 | 61 | 2 | 10 | 52 | 13 | 46 | 58 | 26 | 20 | 11 | 19 | 22 | ***** |
| **346** | | **I** | 14 | 23 | 48 | 49 | 15 | 71 | 48 | 2 | 60 | 6 | 13 | 48 | 24 | 48 | 59 | 25 | 23 | 4 | 29 | 28 | **V** |
| **347** | | **A** | 2 | 23 | 53 | 37 | 23 | 18 | 51 | 30 | 55 | 24 | 20 | 47 | 20 | 50 | 61 | 15 | 22 | 23 | 42 | 41 | ***** |
| **348** | | **G** | 29 | 65 | 71 | 72 | 62 | 1 | 70 | 62 | 78 | 64 | 58 | 47 | 53 | 67 | 78 | 34 | 43 | 71 | 61 | 74 | ***** |
| **349** | | **A** | 2 | 18 | 33 | 30 | 11 | 35 | 23 | 11 | 38 | 12 | 13 | 24 | 8 | 21 | 32 | 12 | 14 | 10 | 14 | 14 | ***** |
| **350** | | **L** | 31 | 41 | 75 | 69 | 27 | 79 | 70 | 12 | 78 | 3 | 14 | 70 | 33 | 68 | 76 | 46 | 40 | 17 | 53 | 52 | ***** |
| **351** | | **F** | 26 | 49 | 65 | 53 | 2 | 74 | 49 | 22 | 72 | 18 | 21 | 61 | 43 | 48 | 70 | 32 | 39 | 27 | 25 | 9 | ***** |
| **352** | | **D** | 23 | 43 | 2 | 11 | 44 | 51 | 27 | 39 | 51 | 40 | 35 | 19 | 21 | 21 | 55 | 19 | 22 | 36 | 43 | 39 | ***** |
| **353** | | **V** | 11 | 20 | 29 | 29 | 13 | 43 | 18 | 5 | 31 | 8 | 10 | 25 | 12 | 21 | 32 | 17 | 13 | 3 | 14 | 12 | ***** |
| **354** | | **H** | 13 | 22 | 12 | 13 | 9 | 27 | 1 | 19 | 19 | 14 | 16 | 4 | 12 | 11 | 16 | 8 | 13 | 17 | 11 | 6 | **N** |
| **355** | | **I** | 13 | 16 | 21 | 27 | 12 | 24 | 16 | 2 | 28 | 7 | 8 | 18 | 7 | 22 | 28 | 12 | 12 | 4 | 18 | 11 | **L** |
| **356** | | **E** | 11 | 24 | 7 | 2 | 14 | 29 | 8 | 17 | 18 | 13 | 16 | 11 | 6 | 7 | 18 | 9 | 13 | 14 | 15 | 12 | ***** |
| **357** | | **A** | 2 | 16 | 22 | 19 | 8 | 27 | 17 | 14 | 27 | 15 | 14 | 20 | 8 | 19 | 23 | 9 | 13 | 15 | 14 | 12 | **F** |
| **358** | | **P** | 35 | 63 | 58 | 54 | 60 | 70 | 58 | 57 | 67 | 58 | 46 | 62 | 2 | 58 | 73 | 43 | 40 | 47 | 51 | 59 | ***** |
| **359** | | **I** | 13 | 17 | 39 | 38 | 6 | 44 | 34 | 2 | 43 | 4 | 7 | 31 | 10 | 34 | 41 | 21 | 17 | 5 | 17 | 9 | **L** |
| **360** | | **Y** | 38 | 56 | 68 | 62 | 8 | 56 | 27 | 25 | 70 | 23 | 26 | 60 | 41 | 55 | 64 | 43 | 37 | 32 | 10 | 2 | ***** |
| **361** | | **M** | 23 | 32 | 57 | 52 | 16 | 66 | 47 | 13 | 55 | 10 | 3 | 47 | 20 | 38 | 44 | 28 | 26 | 13 | 21 | 23 | ***** |

**Table ST1. *Cont.***

| ***Sau* NorAI** | | **Alternative aminoacid** | | | | | | | | | | | | | | | | | | | | ***Sep***  **NorA** |
| --- | --- | --- | --- | --- | --- | --- | --- | --- | --- | --- | --- | --- | --- | --- | --- | --- | --- | --- | --- | --- | --- | --- |
|  |  | **A** | **C** | **D** | **E** | **F** | **G** | **H** | **I** | **K** | **L** | **M** | **N** | **P** | **Q** | **R** | **S** | **T** | **V** | **W** | **Y** |  |
| **362** | **A** | 3 | 17 | 53 | 48 | 31 | 29 | 52 | 20 | 60 | 28 | 28 | 40 | 18 | 49 | 62 | 17 | 23 | 21 | 30 | 41 | ***** |
| **363** | **I** | 15 | 16 | 58 | 53 | 21 | 42 | 55 | 3 | 64 | 12 | 15 | 47 | 27 | 55 | 65 | 23 | 21 | 8 | 47 | 32 | ***** |
| **364** | **G** | 12 | 28 | 45 | 47 | 25 | 3 | 43 | 14 | 51 | 27 | 33 | 30 | 16 | 45 | 53 | 19 | 31 | 24 | 31 | 45 | **A** |
| **365** | **V** | 19 | 28 | 65 | 57 | 24 | 62 | 52 | 8 | 73 | 13 | 18 | 65 | 21 | 59 | 74 | 38 | 30 | 3 | 54 | 43 | ***** |
| **366** | **S** | 5 | 8 | 18 | 15 | 10 | 24 | 20 | 10 | 29 | 9 | 4 | 14 | 9 | 16 | 35 | 2 | 8 | 11 | 21 | 17 | ***** |
| **367** | **L** | 29 | 35 | 72 | 63 | 23 | 71 | 65 | 12 | 76 | 3 | 15 | 66 | 42 | 64 | 72 | 50 | 39 | 19 | 50 | 45 | ***** |
| **368** | **A** | 2 | 10 | 36 | 31 | 7 | 28 | 34 | 8 | 45 | 10 | 12 | 27 | 9 | 31 | 44 | 12 | 12 | 8 | 27 | 27 | **S** |
| **369** | **G** | 17 | 33 | 56 | 58 | 43 | 3 | 57 | 41 | 65 | 37 | 43 | 36 | 35 | 59 | 67 | 23 | 38 | 34 | 32 | 50 | ***** |
| **370** | **V** | 11 | 15 | 42 | 37 | 12 | 47 | 40 | 3 | 43 | 8 | 10 | 40 | 14 | 39 | 56 | 19 | 15 | 2 | 15 | 24 | **I** |
| **371** | **V** | 22 | 26 | 63 | 55 | 21 | 52 | 61 | 8 | 65 | 16 | 17 | 63 | 25 | 60 | 74 | 35 | 28 | 3 | 38 | 36 | **I** |
| **372** | **I** | 24 | 23 | 70 | 66 | 18 | 78 | 63 | 3 | 76 | 10 | 17 | 65 | 30 | 66 | 65 | 41 | 31 | 8 | 48 | 31 | ***** |
| **373** | **V** | 11 | 12 | 47 | 41 | 12 | 55 | 40 | 3 | 54 | 7 | 9 | 44 | 16 | 39 | 58 | 17 | 14 | 2 | 16 | 18 | **I** |
| **374** | **L** | 19 | 22 | 54 | 48 | 5 | 73 | 42 | 8 | 47 | 2 | 8 | 45 | 16 | 33 | 38 | 25 | 23 | 11 | 13 | 15 | **F** |
| **375** | **I** | 13 | 24 | 44 | 36 | 9 | 44 | 26 | 2 | 45 | 6 | 9 | 39 | 17 | 39 | 29 | 20 | 17 | 4 | 18 | 14 | ***** |
| **376** | **E** | 10 | 23 | 9 | 2 | 15 | 32 | 10 | 17 | 16 | 16 | 13 | 13 | 8 | 8 | 17 | 8 | 12 | 11 | 10 | 19 | ***** |
| **377** | **K** | 22 | 37 | 40 | 25 | 28 | 52 | 23 | 27 | 3 | 30 | 18 | 19 | 18 | 16 | 9 | 22 | 20 | 27 | 25 | 23 | ***** |
| **378** | **Q** | 6 | 14 | 12 | 6 | 13 | 40 | 7 | 14 | 8 | 9 | 8 | 9 | 6 | 2 | 7 | 8 | 10 | 12 | 14 | 12 | **G** |
| **379** | **H** | 15 | 21 | 15 | 9 | 16 | 32 | 2 | 17 | 13 | 8 | 16 | 10 | 9 | 11 | 10 | 11 | 13 | 18 | 17 | 8 | **L** |
| **380** | **R** | 18 | 27 | 31 | 18 | 24 | 36 | 16 | 23 | 9 | 19 | 17 | 11 | 15 | 10 | 3 | 22 | 17 | 28 | 26 | 19 | **K** |
| **381** | **A** | 3 | 15 | 21 | 15 | 19 | 18 | 20 | 13 | 17 | 13 | 12 | 15 | 8 | 14 | 17 | 10 | 12 | 13 | 21 | 19 | **S** |
| **382** | **K** | 10 | 14 | 16 | 10 | 14 | 26 | 10 | 13 | 3 | 14 | 10 | 9 | 9 | 9 | 5 | 12 | 6 | 14 | 14 | 11 | **R** |
| **383** | **L** | 10 | 12 | 23 | 20 | 10 | 31 | 14 | 6 | 17 | 3 | 7 | 16 | 9 | 15 | 9 | 12 | 13 | 8 | 16 | 13 | **R** |
| **384** | **K** | 9 | 11 | 15 | 9 | 10 | 23 | 8 | 9 | 3 | 11 | 8 | 7 | 8 | 8 | 4 | 10 | 9 | 11 | 10 | 9 | ***** |
| **385** | **E** | 6 | 7 | 6 | 4 | 7 | 18 | 6 | 7 | 9 | 7 | 7 | 7 | 4 | 4 | 7 | 6 | 8 | 7 | 7 | 7 | ***** |
| **386** | **Q** | 7 | 9 | 9 | 7 | 8 | 18 | 7 | 8 | 7 | 8 | 7 | 9 | 7 | 6 | 8 | 8 | 9 | 8 | 7 | 7 | **A** |
| **387** | **N** | 5 | 6 | 6 | 6 | 5 | 12 | 5 | 5 | 6 | 5 | 5 | 5 | 5 | 7 | 5 | 7 | 6 | 6 | 5 | 5 | **-** |
| **388** | **M** | 6 | 7 | 8 | 7 | 5 | 11 | 8 | 4 | 8 | 2 | 4 | 7 | 5 | 7 | 8 | 6 | 6 | 4 | 6 | 6 | **-** |
